# Phylogenetic Distribution of CRISPR-Cas Systems in *Staphylococcus lugdunensis*

**DOI:** 10.1128/spectrum.01247-21

**Published:** 2021-12-01

**Authors:** Cheng-Yen Kao, Jang-Jih Lu, Lee-Chung Lin, Hsiao-Chi Lin, Shih-Cheng Chang

**Affiliations:** a Institute of Microbiology and Immunology, School of Life Science, National Yang Ming Chiao Tung University, Taipei, Taiwan; b Department of Laboratory Medicine, Linkou Chang Gung Memorial Hospital, Taoyuan, Taiwan; c Department of Medical Biotechnology and Laboratory Science, Chang Gung University, Taoyuan, Taiwan; d Department of Medicine, College of Medicine, Chang Gung University, Taoyuan, Taiwan; The National University of Singapore and the Genome Institute of Singapore

**Keywords:** CRISPR-Cas system, multilocus sequence typing, oxacillin susceptibility, *Staphylococcus lugdunensis*, spacer sequences

## Abstract

Clustered regularly interspaced short palindromic repeats (CRISPRs) and CRISPR-associated (Cas) genes (CRISPR-Cas) are present in many bacterial genomes with functions beyond adaptive immunity. We aimed to characterize the CRISPR-Cas system in the pathogenic Gram-positive bacterium Staphylococcus lugdunensis and determine its association with sequence types (STs) determined by multilocus sequence typing (MLST) and oxacillin susceptibility. Primers were designed to detect and sequence types IIIA and IIC CRISPR-Cas in 199 *S. lugdunensis* isolates. MLST and oxacillin susceptibility tests were also performed on the isolates. We found that 84 *S. lugdunensis* isolates had type IIIA CRISPR-Cas, while 46 had type IIC. The results showed a strong association between STs and CRISPR-Cas types. The ST1, ST6, ST12, and ST15 isolates had type IIIA CRISPR-Cas systems, and the ST4, ST27, and ST29 isolates had type IIC CRISPR-Cas. Interestingly, of 83 isolates containing type IIIA CRISPR-Cas, 17 (20.5%) were oxacillin-resistant *S. lugdunensis* (ORSL), and all of these ORSL isolates belonged to ST6 cluster 1. Moreover, spacers 23 and 21 were found in 16 and 17 ORSL isolates, respectively. In contrast, all 46 isolates with type IIC CRISPR-Cas were susceptible to oxacillin. Our results showed that 41.3% of CRISPR-Cas IIIA spacers were homologous to plasmids and 20.2% were homologous to phages. However, in type IIC CRISPR-Cas, 11.8% and 39.9% of spacers showed sequence homology with plasmids and phages, respectively. In conclusion, we found that the distribution and composition of the CRISPR-Cas system in *S. lugdunensis* was associated with STs and oxacillin susceptibility.

**IMPORTANCE** CRISPR-Cas systems have been characterized as playing several biological roles in many bacterial genomes. Moreover, CRISPR-Cas systems are useful for epidemiological, diagnostic, and evolutionary studies of pathogenic bacteria. However, the characteristics of CRISPR-Cas systems in Staphylococcus lugdunensis have been rarely reported. In this study, we revealed that type IIIA CRISPR-Cas was dominant in *S. lugdunensis* isolates, followed by type IIC CRISPR-Cas. Moreover, the composition of CRISPR-Cas spacers was strongly associated with multilocus sequence typing and oxacillin susceptibility of *S. lugdunensis*. These results advance our understanding of the evolution of CRISPR-Cas systems; however, the biological functions of CRISPR-Cas systems in *S. lugdunensis* remain to be further characterized.

## INTRODUCTION

Staphylococcus lugdunensis is a Gram-positive, catalase-positive, and coagulase-negative Staphylococcus (CoNS) bacterium. Staphylococcus lugdunensis has emerged as an important pathogen, implicated in clinically invasive infections such as endocarditis and bacteremia in recent years (1). β-lactams, such as oxacillin and methicillin, are commonly prescribed for the treatment of *S. lugdunensis* infections; however, oxacillin-resistant *S. lugdunensis* (ORSL) has been frequently reported over the last decade (2, 3). Staphylococcus lugdunensis expressing penicillin-binding protein 2a (PBP2a), encoded by the *mecA* gene, shows a lower affinity for β-lactams and is responsible for β-lactam resistance (1). The only known pathway for *mecA* to spread via horizontal gene transfer is for it to be carried on a mobile genetic element called staphylococcal cassette chromosome *mec* (SCC*mec*) (4, 5).

Many genotyping tools have been developed for the epidemiological study of pathogenic bacteria (6–10). For instance, Chassian et al. developed a multilocus sequence typing (MLST) scheme to study the genetic relationships and population structure of *S. lugdunensis* isolates from different clinical and geographic sources, using DNA sequence analysis of seven housekeeping genes: *aroE*, *dat*, *ddl*, *gmk*, *ldh*, *recA*, and *yqiL* (6). They found that 87 *S. lugdunensis* isolates could be defined into 20 sequence types (STs) and 5 clonal complexes (6). Although the isolates recovered from hematogenic infections such as blood and osteoarticular infections, or from skin and soft tissue infections, did not cluster in separate lineages, penicillin-resistant isolates clustered mainly in one clonal complex (6). Based on MLST, Cheng et al. reported that a major endemic clone of ST6 ORSL carrying the SCC*mec* type II was able to persist long-term in a Taiwanese hospital (7).

Clustered regularly interspaced short palindromic repeats (CRISPRs) and CRISPR-associated (Cas) genes (CRISPR-Cas) are present in many bacterial and archaeal genomes (11). CRISPR-Cas systems are classified into two distinct classes based on design principles of the effector modules (3). CRISPR-Cas class 1 is further divided into types I, III, and IV, and class 2 is divided into types II, V, and VI, with each type being characterized by different effector module architectures that include unique signature proteins (3). In addition, the variable spacer content of CRISPR-Cas systems has been used as a complementary typing tool in epidemiological, diagnostic, and evolutionary studies of pathogenic bacteria, such as Salmonella, *Yersinia*, and Staphylococcus (11–13). Rossi et al. reported a low prevalence (15/122, 12.3%) of complete CRISPR-Cas systems found in CoNS, which consisted of mainly class 1 type IIIA and class 2 type IIC systems; this is consistent with a study published by Argemi et. al. (14, 15). However, the association of *S. lugdunensis* CRISPR-Cas systems with MLST STs and antibiotic susceptibility remains unclear. Therefore, in the current study, we aimed to characterize the CRISPR-Cas systems of *S. lugdunensis*.

## RESULTS

### Detection of CRISPR-Cas systems in *S. lugdunensis* isolates.

**FIG 1:**
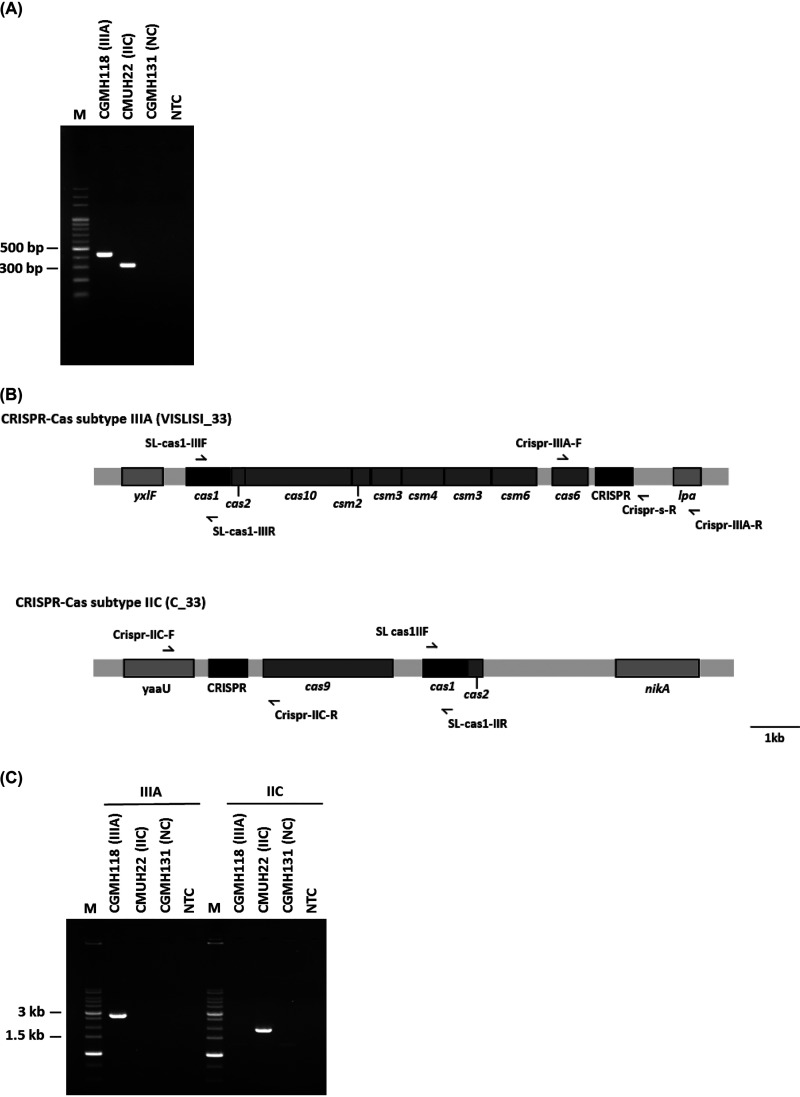
Primer design and evaluation for CRISPR-Cas detection and sequencing. (A) Control CRISPR-Cas types were used to evaluate a multiplex PCR and type IIIA-specific (SL-cas1-IIIF and SL-cas1-IIIR) and type IIC-specific (SL-cas1-IIF and SL-cas1-IIR) primers for the *cas1* gene. (B) Type-specific primers were designed to amplify type IIIA and type IIC *cas1* genes: primer set SL-cas1-IIIF and SL-cas1-IIIR for type IIIA, primer set SL-cas1-IIF and SL-cas1-IIR for type IIC. CRISPR array and flanking regions were amplified and sequenced using primer set Crispr-IIIA-F and Crispr-IIIA-R for type IIIA and primer set Crispr-IIC-F and Crispr-IIC-R for type IIC. Downstream sequencing of type IIIA CRISPR array was performed using the Crispr-s-R primer. (C) CRISPR arrays of two types were amplified separately, using two different primer sets: Crispr-IIIA-F and Crispr-IIIA-R for type IIIA, and Crispr-IIC-F and Crispr-IIC-R for type IIC. PCR products were purified and directly sequenced. M, DNA marker; ORSL CGMH118 (type IIIA CRISPR, 436 bp); ORSL CMUH22 (type IIC CRISPR, 316 bp); ORSL CGMH131 (CRISPR negative control, NC); NTC, no template control.

Class 1 type IIIA and class 2 type IIC CRISPR-Cas systems have been mainly found in *S. lugdunensis* (14). Considering that the Cas1 protein is present in these two types of CRISPR-Cas systems, we designed primers to specifically detect the *cas1* gene and then evaluated 199 *S. lugdunensis* isolates (Fig. 1A and B). Whole-genome sequences were determined for type IIIA strain CGMH118, type IIC strain CMUH22, and CRISPR-Cas-negative strain CGMH131 (unpublished data), and these strains were used as Cas1-PCR (PCR) controls (Fig. 1A). We collected 49 ORSL and 150 oxacillin-susceptible *S. lugdunensis* (OSSL) isolates and determined the distribution of CRISPR-Cas in the isolates. The PCR results showed that 130/199 (65.3%) of the *S. lugdunensis* isolates had CRISPR-Cas systems, including 84 (42.2%) with type IIIA and 46 (23.1%) with type IIC (Table 1). MLST of the 199 isolates revealed 11 different MLST types (Table 1). The most prevalent ST was ST6 (66/199, 33.2%), followed by ST3 (60/199, 30.2%) and ST27 (26/199, 13.1%) (Table 1). Type IIIA CRISPR-Cas was found in ST1, ST6, ST12, and ST15, and type IIC CRISPR-Cas was found in ST4, ST27, and ST29 (Table 1). No type IIIA or type IIC CRISPR-Cas were identified in ST2, ST3, ST9, or ST24 (Table 1). To characterize the spacer sequences of the 130 CRISPR-Cas-positive isolates, we designed primers for spacer amplification and sequencing (Fig. 1B). The specificity of the primers used to amplify the spacers of the type IIIA and type IIC CRISPR-Cas-positive isolates is shown in Fig. 1C. The spacers of one ST1 isolate with a type IIIA CRISPR-Cas could not be amplified by PCR and were therefore excluded from further analysis (Table 1).

**TABLE 1:** Distribution of oxacillin susceptibility, STs, and CRISPR-Cas systems among 199 Staphylococcus lugdunensis isolates

Oxacillin susceptibility	MLST	No. of isolates (%) (*n* = 199)	CRISPR-Cas types, *n* (%)	
			IIIA (*n* = 84)	IIC (*n* = 46)
ORSL^*a*^	3	32 (16.1)	0	0
	6	17 (8.5)	17 (20.2)	0
OSSL^*a*^	1	11 (5.5)	11^*b*^ (13.1)	0
	2	3 (1.5)	0	0
	3	28 (14.1)	0	0
	4	14 (7.0)	0	14 (30.4)
	6	49 (24.6)	49 (58.3)	0
	9	4 (2.0)	0	0
	12	4 (2.0)	4 (4.8)	0
	15	3 (1.5)	3 (3.6)	0
	24	1 (0.5)	0	0
	27	26 (13.1)	0	26 (56.5)
	29	6 (3.0)	0	6 (13.0)
	Untypable	1 (0.5)	0	0

a ORSL, oxacillin-resistant *S. lugdunensis*; OSSL, oxacillin-susceptible *S. lugdunensis*.

b Spacer content of one type IIIA CRISPR-Cas ST1 isolate could not be amplified by using the primers designed in this study.

### CRISPR-Cas spacer content was associated with STs and oxacillin susceptibility.

**FIG 2:**
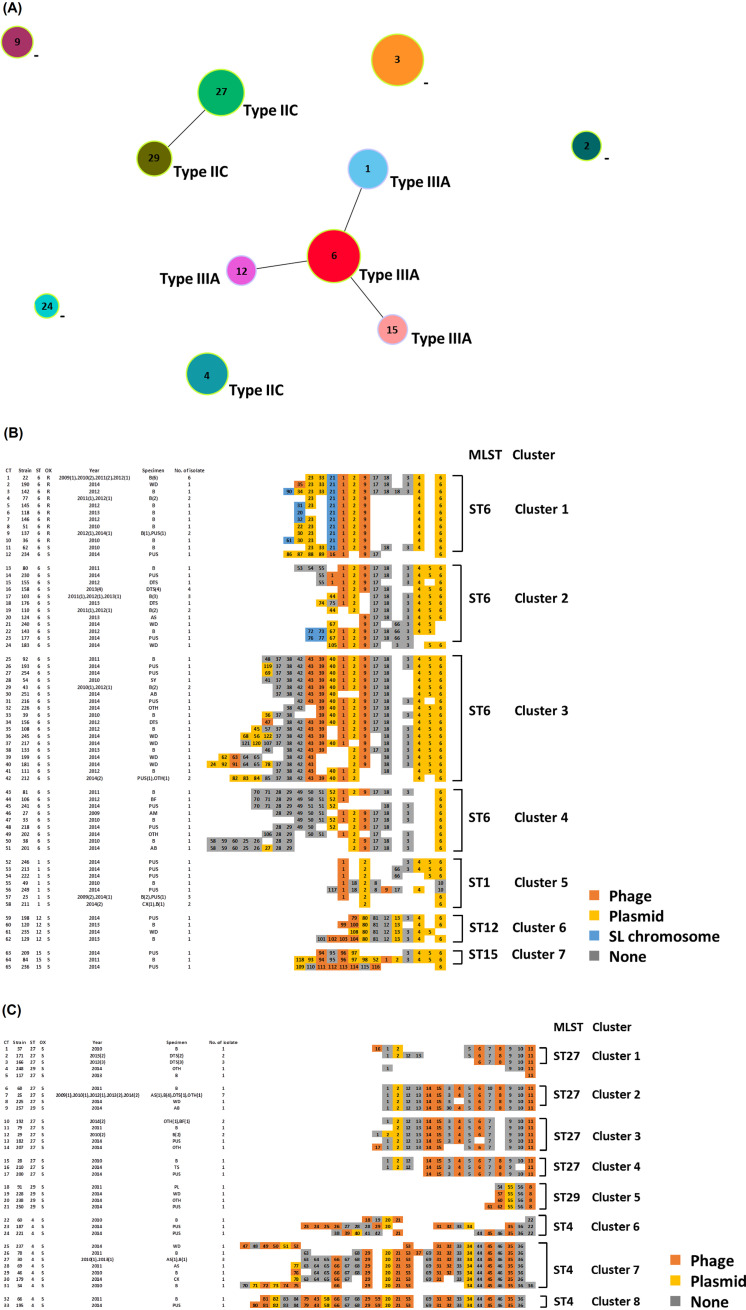
Phylogenetic analysis of MLST and CRISPR spacer content comparison in *S. lugdunensis*. (A) Phylogenetic relationships of the STs associated with types of CRISPR-Cas systems are shown in different colored circles. The eBURST diagram was generated using PHYLOViZ with the goeBURST algorithm. STs are grouped according to the similarity of their allelic profiles. “-” indicates STs that contain no CRISPR-Cas systems. Each circle filled with different colors denotes a particular ST. Circle sizes are proportional to the number of isolates in the STs. The yellow border color indicates predicted founder STs. (B, C) Spacer content comparison of *S. lugdunensis* strains containing type IIIA CRISPR (panel B) and type IIC CRISPR (panel C). Only spacers, and not the direct repeats, are shown in panels B and C. Each spacer is indicated as a specific number. CRISPR type is represented as a combination of spacer numbers and a background color. The colors corresponding to spacer sequence sources in the NCBI BLAST analysis indicate phage (orange), plasmid (yellow), *S. lugdunensis* chromosome (blue), and none (gray). CRISPR type (CT), strain name, sequence type (ST), oxacillin (OX) susceptibility, year, specimen type, and number of isolates are indicated. Values in brackets indicate the number of isolates. CTs belonging to specific MLSTs or their clusters are indicated on the right. AB, abscess; AM, amniotic fluid; AS, ascites; B, blood; BF, body fluid; CX, endocervix discharge; DTS, deep tissue; OTH, other; PUS, pus; PL, pleural effusion; SY, synovial fluid; TS, tissue; WD, wound.

**FIG 3:**
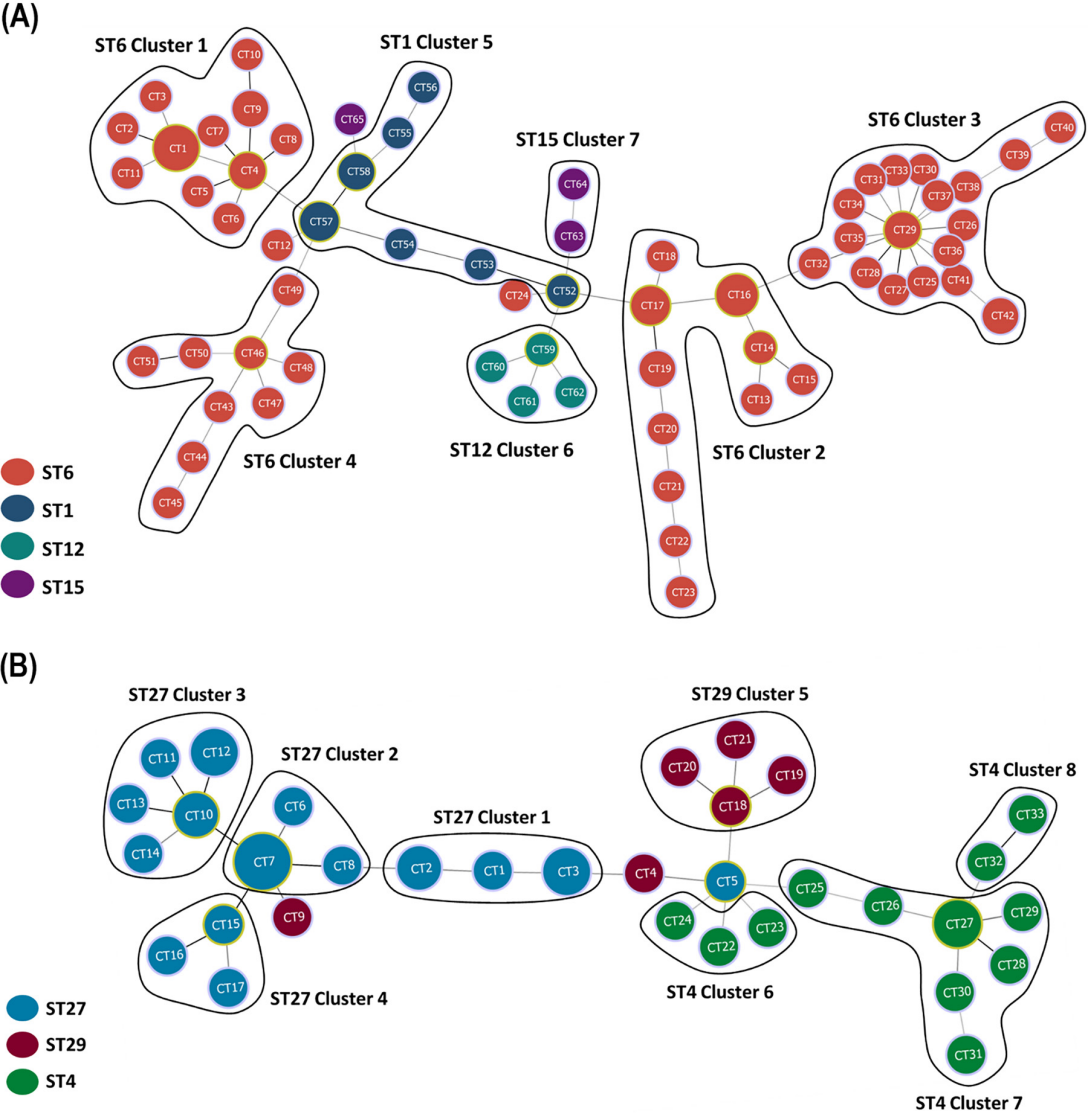
Minimum spanning tree of CTs for *S. lugdunensis* strains having type IIIA (panel A) and type IIC (panel B) CRISPR-Cas systems according to STs. Each node represents one CT, with the number in the circle representing the specific CT. Circle sizes are proportional to the number of isolates within the CTs. CT profiles were grouped by STs. (A) ST6 strains were further divided into four clusters (1–4). Different STs are marked with corresponding colors as indicated. (B) ST4 and ST27 strains were further divided into three (6–8) and four clusters (1–4), respectively. Different STs are marked with corresponding colors as indicated.

To further validate the association between CRISPR-Cas types and STs, we performed a phylogenetic analysis based on the sequences of seven housekeeping genes for MLST. The results revealed a strong association between ST1, ST6, ST12, and ST15 (i.e., descended from a single-locus variant genotype), which had type IIIA CRISPR-Cas systems (Fig. 2A). Among type IIC CRISPR-Cas-positive isolates, ST27 and ST29 (i.e., also descended from a single-locus variant genotype) exhibited strong ancestral relatedness, but ST4 did not (Fig. 2A). We then performed a phylogenetic analysis based on type IIIA and type IIC CRISPR-Cas spacer content to investigate the association between STs, oxacillin susceptibility, and CRISPR-Cas spacer sequences (Fig. 2B and C). The detailed homologs and information regarding the spacers identified in type IIIA and IIC CRISPR-Cas systems are provided in the supplementary data (Tables S4 and S5). In total, 80 isolates had type IIIA CRISPR-Cas, comprising seven clusters and 65 CRISPR types (CTs) (Fig. 2B); however, three isolates with type IIIA CRISPR-Cas could not be classified into any clusters (Fig. 2B and 3A). Spacer 6 was dominant in type IIIA CRISPR-Cas-containing isolates (77/83, 92.8%), followed by spacer 2 (70/83, 84.3%). Of the 83 isolates containing type IIIA CRISPR-Cas, 17 (20.5%) were resistant to oxacillin. All of these ORSL isolates belonged to ST6 cluster 1 (Fig. 2B). Moreover, spacer 23, which showed homology with a Staphylococcus aureus strain 16405 plasmid, was found in 16 of the ORSL isolates, and spacer 21, which showed homology with the *S. lugdunensis* chromosome, was found in 17 of the ORSL isolates (Fig. 2B and Table S4 in the supplemental material). Spacers 9, 17, and 18 were highly conserved in ST6 clusters 1–4 (Fig. 2B). Spacer 9 showed homology with Staphylococcus 187-like phages in the *S. lugdunensis* genome, but we were unable to determine the homologs of spacers 17 and 18 (Table S4). Most ST1 and ST6 clusters (clusters 1 to 5) had spacers 1 and 2, which showed homology with Campylobacter phage CP220 and Clostridium botulinum strain CDC_67071 plasmid pNPD7, respectively. All ST15 cluster 7 isolates had spacers 94, 95, 96, and 97, of which 94 and 96 showed homology with phages in the *S. lugdunensis* genome (Fig. 2B and Table S4). Spacers 12, 13, 80, and 81 were highly conserved in the ST12 cluster 6 isolates (Fig. 2B). Spacer 13 showed homology with S. aureus plasmid pNTUH_3874, and spacer 80 showed homology with a Staphylococcus warneri strain FDAARGOS_754 plasmid (Table S4). We were unable to determine the homologs of spacers 12 and 81.

The mean number of spacers in *S. lugdunensis* isolates containing type IIIA CRISPR-Cas systems was 10.2 (818 spacers/80 isolates) (Table 2). We found that isolates belonging to ST6 cluster 3 contained the most spacers (mean, 14.3 spacers/strain), and strains that belonged to ST1 cluster 5 contained the fewest spacers (mean, 4.5 spacers/strain) (Table 2). Moreover, only strains in ST6 cluster 1 and ST6 cluster 2 had spacers showing homology with the *S. lugdunensis* chromosome (Table 2). Overall, 338 (41.3%) spacers in the type IIIA CRISPR-Cas system showed homology with plasmids, and 165 (20.2%) showed homology with phages. Moreover, 59.1% of the spacers identified in the strains that belonged to ST15 were homologous to plasmids, while 24.9% of the spacers identified in strains belonging to ST6 cluster 3 were homologous to phages (Table 2).

**TABLE 2:** Phylogenetic clusters, number of spacers, and summary of spacer sequence matches of the type IIIA CRISPR-Cas system to the NCBI nonredundant database

ST	Cluster	No. of isolates^*a*^	No. of spacers			No. (%) of spacers matched with genomic homologs			
			Total^*b*^	Mean	Min–max	Phage	Plasmid	SL chr^*c*^	None^*d*^
ST6	1	18	172	9.6	7–14	37 (21.5)	85 (49.4)	23 (13.4)	27 (15.7)
	2	17	153	9	7–12	26 (17.0)	69 (45.1)	4 (2.6)	54 (35.3)
	3	20	285	14.3	11–17	71 (24.9)	108 (37.9)	0 (0)	106 (37.2)
	4	9	106	11.8	10–15	11 (10.4)	22 (20.8)	0 (0)	73 (68.9)
ST1	5	10	45	4.5	2–9	9 (20.0)	24 (53.3)	0 (0)	12 (26.7)
ST12	6	4	35	8.8	8–11	6 (17.1)	17 (48.6)	0 (0)	12 (34.3)
ST15	7	2	22	11	8–14	5 (22.7)	13 (59.1)	0 (0)	4 (18.2)
Total		80	818	10.2	2–17	165 (20.2)	338 (41.3)	27 (3.3)	288 (35.2)

a Three isolates containing type IIIA CRISPR-Cas could not be classified into any CTs or clusters and were not included in the table (Fig. 2B).

b Total number of spacers for all isolates in each cluster.

c SL chr, *S. lugdunensis* chromosome.

d None, spacer sequences were not related to phages, plasmids, or the chromosome of *S. lugdunensis*.

Forty-three *S. lugdunensis* isolates with type IIC CRISPR-Cas comprised 8 clusters and 33 CTs (Fig. 2C). Three isolates with type IIC CRISPR-Cas could not be classified into any clusters (Fig. 2C and 3B). Spacers 1, 2, and 5 to 15 were highly conserved in isolates belonging to ST27 clusters 1 to 4 (Fig. 2C). However, ST27 cluster 1 lacked spacers 3, 4, 14, and 15; cluster 3 lacked spacer 8; and cluster 4 lacked spacer 13 (Fig. 2C). Spacers 6, 8, 11, 14, and 15 showed homology with different phages in the *S. lugdunensis* genome (Table S5). All ST29 isolates had spacers 55 and 56 (Fig. 2C). Spacer 55 showed homology with the *Secundilactobacillus paracollinoides* strain TMW 1.1979 plasmid pL11979-2 (Table S5). We were unable to determine the homologs of spacer 56. Spacers 20, 21, 29, 31, 32 to 36, 44 to 46, and 53 were highly conserved in ST4 isolates, but ST4 cluster 1 lacked spacer 53 (Fig. 2C). Among the ST4-specific spacers, spacers 21, 29, 31, 32, 35, 45, and 53 showed homology with phages in Staphylococcus genomes (Table S5).

All isolates with type IIC CRISPR-Cas were susceptible to oxacillin. The mean number of spacers in *S. lugdunensis* containing type IIC CRISPR-Cas systems was 13.7 (591 spacers/43 isolates) (Table 3), higher than the number in *S. lugdunensis* containing type IIIA CRISPR-Cas systems (mean 10.2 spacers/strain) (Table 2). Our results showed that strains belonging to ST4 cluster 8 contained the highest mean number of spacers (25.5 spacers/strain), while the strains that belonged to ST29 cluster 5 contained the lowest mean number of spacers (4.3 spacers/strain) (Table 3). Moreover, no spacers in type IIC CRISPR-Cas showed homology with the *S. lugdunensis* chromosome (Table 3). Overall, 70 (11.8%) and 236 (39.9%) spacers in the type IIC CRISPR-Cas system showed homology with plasmids and phages, respectively (Table 3). Of spacers identified in strains belonging to ST27 cluster 1, 24% showed homology with plasmids, while 49% of spacers identified in strains of ST4 cluster 8 showed homology with phages (Table 3).

**TABLE 3:** Phylogenetic clusters, number of spacers, and summary of spacer sequence matches of the type IIC CRISPR-Cas system to the NCBI nonredundant database

ST	Cluster	No. of isolates^*a*^	No. of spacers			No. (%) of spacers matched with genomic homologs			
			Total^*b*^	Mean	Min–max	Phage	Plasmid	SL chr^*c*^	None^*d*^
ST27	1	6	50	8.3	6–11	10 (20.0)	12 (24.0)	0 (0)	28 (56.0)
	2	9	134	14.9	14–15	53 (39.6)	9 (6.7)	0 (0)	72 (53.7)
	3	7	97	13.9	13–15	34 (35.1)	9 (9.3)	0 (0)	54 (55.7)
	4	3	38	12.7	11–14	18 (47.4)	2 (5.3)	0 (0)	18 (47.4)
ST29	5	4	17	4.3	4–5	8 (47.1)	4 (23.5)	0 (0)	5 (29.4)
ST4	6	3	33	11	5–16	14 (42.4)	4 (12.1)	0 (0)	15 (45.5)
	7	9	171	19	16–21	74 (43.3)	22 (12.9)	0 (0)	75 (43.9)
	8	2	51	25.5	25–26	25 (49.0)	8 (15.7)	0 (0)	18 (35.3)
Total		43	591	13.7	4–26	236 (39.9)	70 (11.8)	0	285 (48.2)

a Three isolates containing type IIC CRISPR-Cas could not be classified into any CTs or clusters and were not included in the table (Fig. 2C).

b Total number of spacers for all isolates in each cluster.

c SL chr, *S. lugdunensis* chromosome.

d None, spacer sequences were not related to phages, plasmids, or the chromosome of *S. lugdunensis*.

## DISCUSSION

Variation in CRISPR-Cas spacer composition and content has been reported in epidemiological, diagnostic, and evolutionary studies of pathogenic bacteria (11, 12). In this study, we characterized the CRISPR-Cas systems in *S. lugdunensis*. We found 84 *S. lugdunensis* isolates with type IIIA CRISPR-Cas and 46 isolates with type IIC (Table 1). Although type IIIA and type IIC CRISPR-Cas are the main types found in *S. lugdunensis* (14), whether other types of CRISPR-Cas are present in *S. lugdunensis* ST2, ST3, ST9, and ST24 remains to be investigated using whole-genome sequencing.

Our results demonstrated that CRISPR-Cas spacer sequences were strongly associated with MLST and oxacillin susceptibility. Previous studies have shown that pandemics caused by multidrug-resistant Klebsiella pneumoniae are mostly due to the global dissemination of high-risk clonal complex 258 (CC258) and related IncF epidemic plasmids (3). The absence of type I-E CRISPR-Cas systems in K. pneumoniae CC258 is strongly associated with the dissemination of IncF epidemic plasmids (3, 16). In Escherichia coli, the type I-F CRISPR-Cas system is more likely to be found in antimicrobial-susceptible E. coli due to the CRISPR system potentially interfering with the acquisition of antimicrobial resistance plasmids, thereby maintaining the susceptibility of these isolates (2). Here, we found that all CRISPR-Cas-positive ORSL isolates belonged to ST6 cluster 1 (Fig. 2B). In addition, spacer 23 of type IIIA CRISPR-Cas was found in 16 ORSL isolates, and spacer 21 of type IIIA CRISPR-Cas was found in 17 ORSL isolates (Fig. 2B). The homologs of spacers 23 and 21 in type IIIA CRISPR-Cas were plasmids and the *S. lugdunensis* chromosome, respectively (Table S4). Surprisingly, spacer 23 showed homology with a S. aureus strain 16405 plasmid that expressed PBP2A encoded by *mecA*. Therefore, the activity of CRISPR-Cas systems in plasmid acquisition and their association with oxacillin susceptibility of *S. lugdunensis* merits further investigation.

Many genotyping tools have been developed for the epidemiological study of pathogenic bacteria, including MLST, which has been considered the gold standard of bacterial strain typing over the last decade (6–10). More recently, Dahyot et al. developed the first two variable number tandem repeat (VNTR)-based schemes for *S. lugdunensis* typing, including a classical length-based multiple loci VNTR analysis (MLVA) method and a sequence-based MLVA method known as the tandem repeat sequence typing (TRST) method (8). Sequences of the alleles of the seven VNTRs were used to determine their internal variability and size homoplasy, and thus, TRST was performed (8). DNA sequence analysis of the repeat-coding region (R domain) of the gene encoding the fibrinogen-binding protein Fbl (*fbl*-typing) has also been used for *S. lugdunensis* typing (9). Among these typing methods, TRST and *fbl*-typing are more discriminatory than MLST for molecular epidemiological studies of *S. lugdunensis* (8, 9). Compared to TRST and *fbl*-typing, which are sequence-based methods for *S. lugdunensis* typing, PCR-based CRISPR-spacer-specific typing is cost-effective and time-saving. However, the efficiency and accuracy of CRISPR-spacer-specific typing vis-à-vis TRST and *fbl*-typing remain to be evaluated.

Karah et al. performed a comparative analysis of CRISPR-based STs to determine the genetic relatedness and track patterns of descent among 187 Acinetobacter baumannii global clone 1 (GC1) isolates (17). They demonstrated that the most recent common ancestor of the currently known GC1 population carried an array of 54 spacers, and that CRISPR-based subtyping is a convenient method for tracing the evolutionary history of particular bacterial clones as a complement to the evolutionary inferences made using their MLST types (17). Consistent with the report of Karah et. al., our current results showed that CRISPR-Cas spacer sequences were associated with MLST (Fig. 2 and 3). Eighty isolates with type IIIA CRISPR-Cas comprised 7 clusters and 65 CTs, and 43 isolates with type IIC CRISPR-Cas comprised 8 clusters and 33 CTs (Fig. 2B and 3A). Moreover, ST6 *S. lugdunensis* isolates were further divided into 4 clusters based on the composition of their CRISPR spacers, and only ST6 cluster 1 was associated with oxacillin resistance (Fig. 2B). In conclusion, our results showed that type IIIA CRISPR-Cas was dominant in the *S. lugdunensis* isolates. Moreover, the composition of the CRISPR-Cas spacers was associated with MLST types and oxacillin susceptibility.

## MATERIALS AND METHODS

### Isolation and identification of *S. lugdunensis*.

A total of 199 *S. lugdunensis* isolates collected from 2009 to 2014 from Chang Gung Memorial Hospital (Taoyuan, Taiwan) were included in the study. All information associated with the 199 isolates (e.g., specimen type and year of isolation) was collected. Staphylococcus lugdunensis isolates were initially identified by Gram staining, biochemical methods (catalase-positive, coagulase-negative, pyrrolidonyl arylamidase-positive, and ornithine decarboxylase-positive results), and rapid PCR detection (18). All the *S. lugdunensis* isolates were also confirmed using a matrix-assisted laser desorption ionization-time of flight mass spectrometry system (MALDI-TOF MS; Bruker Biotyper, database 2.0). The isolates were stored in tryptic soy broth containing 20% glycerol at −80°C until use.

### CRISPR-Cas system identification and sequencing.

The Crispr-IIIA-F/Crispr-IIIA-R and Crispr-IIC-F/Crispr-IIC-R primers were designed to specifically detect the *cas1* gene for CRISPR-Cas types IIIA and IIC, respectively (Fig. 1B and Table S1). The primers were designed based on *S*. *lugdunensis* whole-genome sequences deposited in the National Center for Biotechnology Information (NCBI) database, including sequences for type IIIA-positive strain VISLISI_33 (accession no. CP020769.1) and type IIC-positive strain C_33 (accession no. CP020768.1). The PCRs were carried out in a total volume of 20 μl containing 2× master mix (Promega, Madison, WI, United States), 10 pmol of each primer, and 1 μl DNA template. The PCR cycling conditions for CRISPR-Cas system detection were 95°C for 3 min; 30 cycles of 30 s at 95°C, 30 s of annealing at 50°C, and 1 min of extension at 72°C; and a final extension for 3 min at 72°C.

The primers Crispr-IIIA-F/Crispr-IIIA-R and Crispr-IIC-F/Crispr-IIC-R were designed for also determining type IIIA and type IIC CRISPR-Cas spacer sequences, respectively (Fig. 1B and Table S2). The PCR cycling conditions for CRISPR-Cas system spacer typing were 95°C for 10 s; 30 cycles of 1 min at 94°C, 1 min of annealing at 55°C, 1 min of extension at 72°C (45 s for CRISPR-Cas IIC), and a final extension for 10 min at 72°C. The Crispr-s-R primer (Table S2) was used to perform downstream sequencing of the type IIIA CRISPR array.

### Analysis and visualization of CRISPR.

All sequences of the CRISPR arrays were characterized to identify CRISPR arrays and extract spacer sequences using CRISPRFinder web software (19) with default settings. Small CRISPRs with evidence level 1 (with three or fewer spacers) were excluded, and only CRISPR arrays with evidence levels 2 to 4 were retained. Level 1 CRISPRs which contained fewer than three spacers were upgraded to level 4 whenever similar direct repeats were found in a level 4 CRISPR. Each unique spacer was assigned a specific spacer number. The spacer sequences were further analyzed to search for known homologies in the NCBI database using BLASTn with an E value cutoff of 0.1. All matches with a bit score above 40 and a query cover above 80% were selected. Only the top 3 hits were shown if multiple hits were found for a given query. Each unique spacer combination within a CRISPR locus was assigned a CT. A new CT number was assigned for CTs not described previously by Argemi et al. (15). The presence (designated “1”) or absence (designated “0”) of each spacer sequence for each strain was summarized using a binary code pattern. The binary patterns of all isolates were uploaded to Phyloviz v. 2.0 to generate a minimum spanning tree using the goeBURST algorithm (20).

### MLST analysis.

MLST was performed for all *S. lugdunensis* isolates according to a previous study (6). Seven housekeeping genes of each isolate, including *aroE*, *dat*, *ddl*, *gmk*, *ldh*, *recA*, and *yqiL*, were PCR-amplified and sequenced using the primers described in Table S3. The sequence data were imported to the *S. lugdunensis* MLST database (http://www.mlst.net) for ST type determination. Sequence types were identified based on the allele profiles. Further analysis was performed using eBURST (http://eburst.mlst.net) to identify clonal complexes (CCs) and founders, as well as to determine the overall population structures. A minimum-evolution (ME) tree of the concatenated sequences (*aroE*-*dat*-*ddl*-*gmk*-*ldh*-*recA*-*yqiL*) for each ST shared by the *S. lugdunensis* isolates was generated using Mega X and the Kimura two-parameter model to estimate genetic distances. Statistical support of the nodes in the ME tree was assessed by performing 1,000 bootstrap resamplings.

### Antimicrobial susceptibility testing.

Susceptibility of the *S. lugdunensis* isolates to oxacillin was determined using an agar dilution assay and interpreted according to the Clinical and Laboratory Standards Institute guidelines (21). Staphylococcus lugdunensis isolates with a MIC *≥* 4 μg/mL were defined as resistant. Staphylococcus aureus ATCC 29213 was used as a control strain. Antimicrobial susceptibility testing was performed in duplicate to ensure reproducibility.

### SCC*mec* typing and sequencing.

All *S. lugdunensis* isolates were subjected to SCC*mec* typing and *mecA* detection using a multiplex PCR assay to amplify the *ccr* and *mec* complexes according to a previous study (22).
