# Supplementary material for: Phylogenetic Distribution of CRISPR-Cas Systems in Staphylococcus lugdunensis
Source: Microbiol Spectr. 2021 Dec 1;9(3):e01247-21. doi: 10.1128/spectrum.01247-21 (PMC8635126; doi:10.1128/spectrum.01247-21)
Supplement: SUPPLEMENTAL FILE 1 — Supplemental material. Download SPECTRUM01247-21_Supp_1_seq7.pdf, PDF file, 0.4 MB [file spectrum01247-21_supp_1_seq7.pdf]

Supplemental materials

Table S1 PCR primers used in CRISPR-Cas detection

CRISPR-Cas type	Primer name	Sequence (5′-3′)
IIIA	SL-cas1-IIIF	TCGAGATTTATACGGCCCACA
	SL-cas1-IIIR	TCAATTCCCCACCTCAATCATT
IIC	SL-cas1-IIF	ACATCACGCTTACTTGTCGA
	SL-cas1-IIR	ACTTTGCAGCTTGTCCTTCA

Table S2 Primers used in CRISPR array sequencing

CRISPR-Cas type	Primer name	Sequence (5′-3′)
IIIA	Crispr-IIIA-F	TCTCCCTTACATGGGGAGCA
	Crispr-IIIA-R	ACCCATGGACTCATGCCAAG
	Crispr-s-R	GCAGAGTTAGCGCTTTGGTT
IIC	Crispr-IIC-F	GCTGCAGTAGGCCTTACCAA
	Crispr-IIC-R	ACAAACGTACACCTGCGTCT

Table S3 Primers used in MLST typing

Gene	Primer	Sequence (5′ to 3′)	size (bp)
aroE	aroE F	5′- ATCGGAGATCCGATTTCACATTC -3′	460
	aroE R	5′- GGCGTTGTATTAATTATAATATC -3′	
dat	dat F	5′- TCGTGGTTATGTTTTTGGTGACGGT -3′	412
	dat R	5′- CTATGAGAAGTAAAGCCAGGAAT -3′	
ddl	ddl F	5′- AGTGCGGAGCACGACGTTTCA -3′	420
	ddl R	5′- ACACTTGATCCCAAATTCGCCGGT -3′	
gmk	gmk F	5′- ATAGTTCTTTCCGGACCATC -3′	431
	gmk R	5′- TCATTGACTACAACGTAATCATA -3′	
ldh	ldh F	5′- ACTTGCAGGTGCCACGTCGA -3′	414
	ldh R	5′- GCTACGCATTTGCAATGGTAACGCA -3′	
recA	recA F	5′- GCACGGCCACCAGGTGTTGT -3′	463
	recA R	5′- AGGCCGTCGCGTATCTAGTGT -3′	
yqiL	yqiL F	5′-GTGCTAAACGCACACCAATTGGA-3′	450
	yqiL R	5′-CTTCAGCATCGATTAGAGGCAC-3′	

Table S4. Sequence matches of spacer from type IIIA CRISPR-Cas systems to NCBI non-redundant database

Spacer	Sequence	BLAST match with known MGEs	Query Cover	Identity %	Max Score	E-value	Accession no.	Location	MGE	Reference
1	TTCTGTATTTCTATTAACCAATTCATAAGTGTCATTA	Campylobacter phage CP220	81%	90%	41.9	0.67	FN667788.1	MGE	Phage	S1, as desccribed by Argemi et al (1)
2	TTCATCATATCCTTTTATATAGTTTTTGTTTTGTT	Clostridium botulinum strain CDC 67071 plasmid pNPD7	74%	92%	39.2	1.4	CP013241.1	MGE	Plasmid	S2, as desccribed by Argemi et al (1)
3	CCAGTTGCTATTTTATTTGTCAACCATTTTATTAA	None	NA	NA	NA	NA	NA	None	NA	S3, as desccribed by Argemi et al (1)
4	TAGAATGTTTAAACAAGGTGTTTCAAAACCTG	Lactobacillus amylolyticus strain L5 plasmid p1	78%	92%	37.4	4.9	CP031836.1	MGE	Plasmid	S4, as desccribed by Argemi et al (1)
5	AACATTTTATTTACGTCTGTTTTTGCCTCTACTAAGTA	Bacillus thuringiensis serovar israelensis strain AM65-52 plasmid pAM65-52-2-350K	87%	85%	40.1	3.3	CP013277.1	MGE	Plasmid	S5, as desccribed by Argemi et al (1)
6	TCGCTATTCAATAAGTTTTGTATTTGTTGTCTCATT	Lactobacillus salivarius strain ZLS006 plasmid unnamed2	77%	89%	38.3	6.5	CP020860.1	MGE	Plasmid	S6, as desccribed by Argemi et al (1)
8	CCAAATAATACTATTAATTTATCTGTTAATGCTGTGG	None	NA	NA	NA	NA	NA	None	NA	S8, as desccribed by Argemi et al (1)
9	TGGTAAATCTTCTACACTGTCGACTGGGTCTTCTA	Staphylococcus lugdunensis SL13 prophage (Staphylococcus phage 187-like)	100%	100%	64.4	7.00E-08	CP041722.1	MGE	Phage	
		Staphylococcus lugdunensis SL55 prophage (Staphylococcus phage phiETA2-like)	100%	100%	64.4	7.00E-08	CP041724.1	MGE	Phage	
		Staphylococcus lugdunensis SL118 prophage (Staphylococcus phage StB12-like)	100%	100%	64.4	7.00E-08	CP041726.1	MGE	Phage	
10	TGTTCCAATTAGCTAAAGATGTTATGGACGGCAAGA	None	NA	NA	NA	NA	NA	None	NA	S10, as desccribed by Argemi et al (1)
12	GAATTTCACTTGCGAATTTTAAATAACTATCCCC	None	NA	NA	NA	NA	NA	None	NA	S12, as desccribed by Argemi et al (1)
13	TTATTAGCTATTTCATTATCTATATTATCACTA	Staphylococcus aureus plasmid pNTUH 3874 NTUH 3874	100%	100%	60.8	8.00E-07	LC377538.1	MGE	Plasmid	
		Staphylococcus aureus strain UP 1654 plasmid unnamed	100%	100%	60.8	8.00E-07	CP047806.1	MGE	Plasmid	
		Staphylococcus aureus strain nan_175_F371_ch plasmid unnamed1	100%	100%	60.8	8.00E-07	CP066493.1	MGE	Plasmid	
16	ATAATCAATTGTTAAATACATTTCATTTGGATTAAT	Staphylococcus lugdunensis FDAARGOS 143 prophage (Staphylococcus phage CNPx-like)	100%	100%	66.2	2.00E-08	CP014023.2	MGE	Phage	
17	TGTGCACTTTTACACTATTAGTTTAAATATCTCCA	None	NA	NA	NA	NA	NA	None	NA	S17, as desccribed by Argemi et al (1)
18	CTTTATATACAGTAGTTAATGACTTATGAAAGGTTTC	None	NA	NA	NA	NA	NA	None	NA	S18, as desccribed by Argemi et al (1)
20	AATGGTCATTAGAAACAGGTGAAGTCAGAGGGAAA	SL chromosome	NA	NA	NA	NA	NA	SL chromosome	NA	This study
21	CAAAACCGAGTGAATTAGAGTTTTAAACAAGCTTG	SL chromosome	NA	NA	NA	NA	NA	SL chromosome	NA	This study
22	CCTAAACTAAGTAAAGATATTTACTTAGTTTTCT	Staphylococcus caprae strain SY333 plasmid pSY333-41	100%	100%	62.6	2.00E-07	CP051645.1	MGE	Plasmid	This study
		Staphylococcus epidermidis strain SESURV_p4_1553 plasmid unnamed2	100%	100%	62.6	2.00E-07	CP043806.1	MGE	Plasmid	This study
		Staphylococcus capitis strain APC2923 plasmid pJOS 01	100%	100%	62.6	2.00E-07	MN602039.1	MGE	Plasmid	This study
23	CGCGGGAAAATAGAACGTTTTTTTCAGACAGT	Staphylococcus aureus strain 16405 plasmid unnamed	100%	100%	59.0	3.00E-06	CP053353.1	MGE	Plasmid	This study
		Staphylococcus aureus strain UP_274 plasmid unnamed	100%	100%	59.0	3.00E-06	CP047853.1	MGE	Plasmid	This study
		Staphylococcus aureus strain UP 883 plasmid unnamed	100%	100%	59.0	3.00E-06	CP047836.1	MGE	Plasmid	This study
24	ATTGGTTATGCGTAGATGGTTAAAAGATAATTCTA	Staphylococcus caprae strain SY333 plasmid pSY333-7	100%	100%	64.4	3.00E-08	CP051646.1	MGE	Plasmid	This study
25	GATGATAATACCGAATACTGCAATTAGAGTGCCAC	None	NA	NA	NA	NA	NA	None	NA	This study
26	TCATTTTGATATTGAATTCAAATTTGATTTCATTT	None	NA	NA	NA	NA	NA	None	NA	This study
27	AAATAGTAATTGGTTATATATTTCAACTACGATTATT	Staphylococcus epidermidis 14.1.R1 plasmid pHOB2	100%	97%	63.5	3.00E-07	CP018844.1	MGE	Plasmid	This study
28	GTCTATAACAGCTTCACCATAGTTTCTCATTTCATC	None	NA	NA	NA	NA	NA	None	NA	This study
29	TGTAACGGCTACTGGACTATCACCGAATTTTGATTTT	None	NA	NA	NA	NA	NA	None	NA	This study
30	ATACCGATAAAATTTATTGATCATTGAGAAGAACCT	Staphylococcus aureus strain ph2 plasmid pPH2	97%	94%	55.4	5.00E-05	MH785233.1	MGE	Plasmid	This study
		Staphylococcus aureus strain pa3 plasmid pPA3	97%	94%	55.4	5.00E-05	MH785229.1	MGE	Plasmid	This study
		Staphylococcus warneri strain WB224 plasmid pWB224 1	97%	91%	50.9	2.00E-03	CP053471.1	MGE	Plasmid	This study
31	GAATAAGAAGATATAAGCGTTAATGCAAAAAGCAT	SL chromosome	NA	NA	NA	NA	NA	SL chromosome	NA	This study
32	AGAGAATTAAATGTATTAAATGTTGAATCTGCAT	SL chromosome	NA	NA	NA	NA	NA	SL chromosome	NA	This study
33	TCTAAAATCTATACCAACAAATGGTTCATCTAAAAAA	Enterococcus faecalis isolate 28157 4#150 genome assembly, plasmid: 2	83%	94%	48.2	7.00E-03	LR962833.1	MGE	Plasmid	This study
		Enterococcus faecalis isolate 26975_2#23 genome assembly, plasmid: 2	83%	94%	48.2	7.00E-03	LR962677.1	MGE	Plasmid	This study
		Enterococcus faecalis isolate 26975_2#21 genome assembly, plasmid: 2	83%	94%	48.2	7.00E-03	LR962651.1	MGE	Plasmid	This study
34	AAAAGATTGTTATTTCTATATAGTATCAAGACAAGA	Staphylococcus aureus ch8 plasmid pCH8	100%	100%	66.2	2.00E-08	MH785245.1	MGE	Plasmid	This study
		Staphylococcus aureus strain MRSA127 15 plasmid pMRSA127 15	100%	100%	66.2	2.00E-08	MF101921.1	MGE	Plasmid	This study
		Staphylococcus aureus strain SA0409 plasmid pHSSA0409	100%	100%	66.2	2.00E-08	KR870312.1	MGE	Plasmid	This study
35	GCAGCTTTTAGTGACATAGAAGATAAAGCGGTATCTT	Staphylococcus lugdunensis SL13 prophage (Staphylococcus phage 187-like)	100%	100%	69.8	3.00E-09	CP041722.1	MGE	Phage	This study
		Staphylococcus lugdunensis SL55 prophage (Staphylococcus phage phiETA2-like)	100%	100%	69.8	3.00E-09	CP041724.1	MGE	Phage	This study
		Staphylococcus lugdunensis SL117 prophage (Staphylococcus phage StB12-like)	100%	100%	69.8	3.00E-09	CP041725.1	MGE	Phage	This study
36	AAATATTTATAATTCATCTATAATTATTACAAAAA	Staphylococcus epidermidis PM221 plasmid 2	100%	97%	59.9	3.00E-06	HG813245.1	MGE	Plasmid	This study
		Staphylococcus epidermidis strain PH1-28 plasmid pSE1	100%	97%	59.9	3.00E-06	CP066375.1	MGE	Plasmid	This study
		Staphylococcus epidermidis strain SESURV_p1_0563 plasmid unnamed1	94%	97%	56.3	3.00E-05	CP043782.1	MGE	Plasmid	This study
37	ACTAATTGGTTTTCATATAGCACTAATATTAACTTA	None	NA	NA	NA	NA	NA	None	NA	This study
38	ACGGACCTTCTTATAAATTAAGTGATTATGGAATTAC	None	NA	NA	NA	NA	NA	None	NA	This study
39	GTTTGTTTTTAAATGGTCTTTGTACTGCAATTCTAA	Staphylococcus lugdunensis SL13 prophage (Staphylococcus phage 187-like)	100%	100%	66.2	2.00E-08	CP041722.1	MGE	Phage	This study
		Staphylococcus lugdunensis SL122 prophage (Staphylococcus phage StB12-like)	100%	100%	66.2	2.00E-08	CP041727.1	MGE	Phage	This study
		Staphylococcus lugdunensis VISLISI_22 prophage (Staphylococcus phage StB12-like)	100%	100%	66.2	2.00E-08	CP020764.1	MGE	Phage	This study
40	TTTATTTTGTTATAAATTAATAATAACACTTATATA	Staphylococcus cohnii strain FDAARGOS_744 plasmid unnamed1	100%	100%	66.2	2.00E-08	CP054808.1	MGE	Plasmid	This study
		Staphylococcus saprophyticus strain UTI-050 plasmid pUTI-050-2	100%	100%	66.2	2.00E-08	CP054577.1	MGE	Plasmid	This study
		Staphylococcus chromogenes strain 1401 plasmid unnamed1	100%	100%	66.2	2.00E-08	CP046029.1	MGE	Plasmid	This study
41	GTTATAAATTCTGTTAAGTTGTTTTTCATATTTTT	None	NA	NA	NA	NA	NA	None	NA	This study
42	GCTTTATTTTGAATAATAGTGTGCATACTTAACCCTA	None	NA	NA	NA	NA	NA	None	NA	This study
43	GCGAAATCGAATAACATTTGTTGAATATGTGGTGGCA	Staphylococcus lugdunensis SL55 prophage (Staphylococcus phage phiETA2-lke)	100%	100%	68	7.00E-09	CP041724.1	MGE	Phage	This study
		Staphylococcus lugdunensis SL117 prophage (Staphylococcus phage StB12-like)	100%	100%	68	7.00E-09	CP041725.1	MGE	Phage	This study
		Staphylococcus lugdunensis SL118 prophage (Staphylococcus phage StB12-like)	100%	100%	68	7.00E-09	CP041726.1	MGE	Phage	This study
44	GCAACTTTTTTTATCGGCTTAAAATTTTGTTTAA	Staphylococcus warneri strain GD01 plasmid p8GD01	100%	100%	62.6	2.00E-07	CP038250.1	MGE	Plasmid	This study
45	TCTATCTTTGCAACAGAACCCTATATTTAATAATATA	Staphylococcus epidermidis strain FDAARGOS_1361 plasmid unnamed2	78%	97%	49.1	0.007	CP070061.1	MGE	Plasmid	This study
		Staphylococcus epidermidis strain Z0118SE0269 plasmid unnamed2	78%	97%	49.1	0.007	CP069217.1	MGE	Plasmid	This study
46	TTTTCTATGTCAATTTTAGATAAAAAATAATCACC	None	NA	NA	NA	NA	NA	None	NA	This study
47	GTAAGTGACGTTAAATTCTTCTTCTATCTTTTGT	Staphylococcus lugdunensis SL117 prophage (Staphylococcus phage StB12-like)	100%	100%	62.6	2.00E-07	CP041725.1	MGE	Phage	This study
		Staphylococcus phage StB20-like	100%	100%	62.6	2.00E-07	KT429161.1	MGE	Phage	This study
48	CCAGATAGTTGTTCCATCCATAAAATATAATCTTACTT	None	NA	NA	NA	NA	NA	None	NA	This study
49	ATAACTTATCTTTTGAGGTGGTGCTGGTAACTTTT	None	NA	NA	NA	NA	NA	None	NA	This study
50	CTCCAAATTCATCATATATATCATAAATGAAATCTTA	None	NA	NA	NA	NA	NA	None	NA	This study
51	CACCAAATTTTGATTTTGTATTAATGAATAATGCTTT	None	NA	NA	NA	NA	NA	None	NA	This study
52	TGGGATGTTATGAATATAATATGGGGTAAAAATC	Staphylococcus aureus strain SCAID OTT1-2021 (597/2) plasmid unnamed	100%	100%	64.4	7.00E-08	CP082814.1	MGE	Plasmid	This study
		Staphylococcus aureus NTUH_3874 plasmid pNTUH_3874-2	100%	100%	64.4	7.00E-08	LC570860.1	MGE	Plasmid	This study
		Staphylococcus equorum strain FDAARGOS_1149 plasmid unnamed1	100%	100%	64.4	7.00E-08	CP068070.1	MGE	Plasmid	This study
53	ACTAGGGGTATATATATAATATGTATAACCTATA	None	NA	NA	NA	NA	NA	None	NA	This study
54	CCTCTTACAATAATAACAGCTTTTGTATTATTTTGT	None	NA	NA	NA	NA	NA	None	NA	This study
55	AATACTTTTAAGAAATATGTTTTGGGATTGTTACC	None	NA	NA	NA	NA	NA	None	NA	This study
56	GACTCATTTCTTGCACATTAGCGACTTACTTTTTG	Staphylococcus epidermidis plasmid SAP105B	100%	97%	59.9	3.00E-06	GQ900453.1	MGE	Plasmid	This study
		Staphylococcus haemolyticus strain SE3.9 plasmid p2	100%	97%	59.9	3.00E-06	CP049093.1	MGE	Plasmid	This study
		Staphylococcus aureus strain AR_0228 plasmid unnamed1	100%	94%	55.4	3.00E-05	CP029661.1	MGE	Plasmid	This study
57	TTATTAATACATTCAATATTATCGACATAATTCCC	None	NA	NA	NA	NA	NA	None	NA	This study
58	CTAAAATTTCGTAAACACTATTAATTTCATTAATT	None	NA	NA	NA	NA	NA	None	NA	This study
59	CGCCGAATTTTGATTTTGTATTGATAAATAACGCTTT	None	NA	NA	NA	NA	NA	None	NA	This study
60	TCAACATTTCCTTAACTGGTTCAAATAACTCTTTCA	None	NA	NA	NA	NA	NA	None	NA	This study
61	ACTAGTATTTGGTGTACTTGCATATTTCTTTGCTAA	SL chromosome	NA	NA	NA	NA	NA	SL chromosome	NA	This study
62	TTACCGTATGACAAATATTAATCAAAAAGTATTAG	Staphylococcus epidermidis strain SESURV_p3_1362 plasmid unnamed1	100%	100%	64.4	7.00E-08	CP043802.1	MGE	Plasmid	This study
		Staphylococcus hominis strain FDAARGOS_748 plasmid unnamed3	100%	100%	64.4	7.00E-08	CP054885.1	MGE	Plasmid	This study
		Staphylococcus epidermidis strain SESURV_p3_0825 plasmid unnamed2	100%	100%	64.4	7.00E-08	CP043794.1	MGE	Plasmid	This study
63	CTCGTGTGGTATAATTAGTTTGTTAATTAAACATGT	Staphylococcus lugdunensis SL13 prophage (Staphylococcus phage 187-like)	100%	100%	66.2	2.00E-08	CP041722.1	MGE	Phage	This study
64	ACTCAAATGATATTTAACTATCTTCCAAATTTTATT	None	NA	NA	NA	NA	NA	None	NA	This study
65	TTGTTGTTGTTCAAAAATACCTCTTAATTCTCCAAAAG	None	NA	NA	NA	NA	NA	None	NA	This study
66	AATGTATTCTCATCTACGTGTACGAACGTCAATCTGTT	None	NA	NA	NA	NA	NA	None	NA	This study
67	AGAAAAGCTTGAAAGCGTCGAAAAGAGAAACGAA	Staphylococcus pettenkoferi strain FDAARGOS_1071 plasmid unnamed	100%	100%	62.6	2.00E-07	CP066063.1	MGE	Plasmid	This study
		Staphylococcus epidermidis strain SESURV_p3_1362 plasmid unnamed1	100%	97%	58.1	1.00E-05	CP043802.1	MGE	Plasmid	This study
		Staphylococcus epidermidis strain SESURV_p3_0825 plasmid unnamed2	100%	97%	58.1	1.00E-05	CP043794.1	MGE	Plasmid	This study
68	ATATTTTTTACAATACAGATTTTTTTCTAAGTCAT	Staphylococcus warneri strain FDAARGOS_754 plasmid unnamed	100%	100%	64.4	7.00E-08	CP054016.1	MGE	Plasmid	This study
		Staphylococcus equorum strain FDAARGOS_1150 plasmid unnamed1	100%	97%	59.9	3.00E-06	CP068064.1	MGE	Plasmid	This study
69	AATAATGTGAATAATCAAGCGCCCACAGTTGAAAGTA	Staphylococcus aureus strain DC.RB_015 plasmid unnamed1	100%	100%	68.0	7.00E-09	CP083435.1	MGE	Plasmid	This study
		Staphylococcus aureus strain ER15890.8A plasmid pER15890_8A.1	100%	100%	68.0	7.00E-09	CP075571.1	MGE	Plasmid	This study
		Staphylococcus aureus strain ER15890.6A plasmid pER15890_6A.1	100%	100%	68.0	7.00E-09	CP075573.1	MGE	Plasmid	This study
70	TTTTGTATTCATAAATATTAATCATAACTTTATCGTT	None	NA	NA	NA	NA	NA	None	NA	This study
71	GAAAAATTAGAATTTCTGGTGTAGGATAGTCACTAT	None	NA	NA	NA	NA	NA	None	NA	This study
72	TTTTTCGAAGCGGGAAATTGCCATATCTTAAAATTTC	SL chromosome	NA	NA	NA	NA	NA	SL chromosome	NA	This study
73	AAAGCAGACATTAATTAATGAAATTAAAACGAGCAT	SL chromosome	NA	NA	NA	NA	NA	SL chromosome	NA	This study
74	ACATGCATACATGACACACGTATACTGTATGCAAC	Staphylococcus aureus CFSAN082783 plasmid pMRSA_24	100%	97%	59.9	3.00E-06	CP031887.1	MGE	Plasmid	This study
		Staphylococcus epidermidis strain FDAARGOS_1363 plasmid unnamed2	100%	97%	59.9	3.00E-06	CP069952.1	MGE	Plasmid	This study
		Staphylococcus epidermidis strain FDAARGOS_1243 plasmid unnamed4	100%	97%	59.9	3.00E-06	CP069477.1	MGE	Plasmid	This study
75	TGGAGAGCTTAACATGTTACAAGAACTATTTAATTTA	None	NA	NA	NA	NA	NA	None	NA	This study
76	TTCCCCAACTTGATTCATATATTGTGAAACTGCATCAC	SL chromosome	NA	NA	NA	NA	NA	SL chromosome	NA	This study
77	ATATATTTAGAAGAATTTAGAAAGGGCTTTAAA	SL chromosome	NA	NA	NA	NA	NA	SL chromosome	NA	This study
78	CGTAAATATTTGTAACCGTTTTATTACCTCTATCC	Staphylococcus haemolyticus strain NY5 plasmid pNY5A	100%	94%	55.4	3.00E-05	CP078160.1	MGE	Plasmid	This study
		Staphylococcus capitis subsp. capitis strain AYP1020 plasmid pAYP1020	88%	94%	48.2	0.005	CP007602.1	MGE	Plasmid	This study
		Staphylococcus simulans bv. staphylolyticus strain NRRL B-2628 plasmid pACK2	100%	89%	46.4	0.017	HQ170520.3	MGE	Plasmid	This study
79	TTAATTATGGATTACTTTAATTTGTACAAAGTTATT	Staphylococcus lugdunensis SL13 prophage (Staphylococcus phage 187-like)	100%	100%	66.2	2.00E-08	CP041722.1	MGE	Phage	This study
		Staphylococcus lugdunensis SL117 prophage (Staphylococcus phage StB12-like)	100%	100%	66.2	2.00E-08	CP041725.1	MGE	Phage	This study
		Staphylococcus lugdunensis SL122 prophage (Staphylococcus phage StB12-like)	100%	100%	66.2	2.00E-08	CP041727.1	MGE	Phage	This study
80	TACAAATTTTTTAAGAAATAATTTTGTTTTTCAAA	Staphylococcus warneri strain FDAARGOS_754 plasmid unnamed	100%	100%	64.4	7.00E-08	CP054016.1	MGE	Plasmid	This study
		Staphylococcus epidermidis strain none genome assembly, plasmid: 3	100%	100%	64.4	7.00E-08	LR735439.1	MGE	Plasmid	This study
		Staphylococcus aureus strain ER03544.3 plasmid unnamed1	100%	100%	64.4	7.00E-08	CP030631.1	MGE	Plasmid	This study
81	GACATAGTTTAAAACTCCTTTATTATTTATTAAT	None	NA	NA	NA	NA	NA	None	NA	This study
82	ACGACATTCGTCGAAACAGTTTAATTAAGGGTTCTTC	Staphylococcus hominis strain FDAARGOS_762 plasmid unnamed4	97%	100%	66.2	2.00E-08	CP054009.1	MGE	Plasmid	This study
		Staphylococcus warneri strain WB224 plasmid pWB224_1	97%	100%	66.2	2.00E-08	CP053471.1	MGE	Plasmid	This study
		Staphylococcus caprae strain SY333 plasmid pSY333-7	97%	100%	66.2	2.00E-08	CP051646.1	MGE	Plasmid	This study
83	GTTTTATTATCTTGATTTTCGTCTATATATTTTGT	Staphylococcus aureus strain SA01 plasmid pSA01-04	100%	100%	64.4	7.00E-08	CP053078.1	MGE	Plasmid	This study
		Staphylococcus epidermidis strain SESURV_p1_0612 plasmid unnamed2	100%	100%	64.4	7.00E-08	CP043786.1	MGE	Plasmid	This study
		Staphylococcus aureus strain SA1159 plasmid pHSSA1159	100%	100%	64.4	7.00E-08	KR870313.1	MGE	Plasmid	This study
84	TACGTCATTGTAGATACAGCTCCTACAGGCCATACCT	Staphylococcus caprae strain SY333 plasmid pSY333-41	100%	100%	68.0	7.00E-09	CP051645.1	MGE	Plasmid	This study
		Staphylococcus pettenkoferi strain FDAARGOS_1071 plasmid unnamed	100%	100%	68.0	7.00E-09	CP066063.1	MGE	Plasmid	This study
85	TTGCTCTAGGCCTTAAATACTTAACACTCAAAAAAAGA	None	NA	NA	NA	NA	NA	None	NA	This study
86	AATGATGAAAATTCACGTATGTCATGGGATCATAA	Staphylococcus aureus VGC1 plasmid pVGC1_1	100%	100%	64.4	7.00E-08	CP039449.1	MGE	Plasmid	This study
		Staphylococcus epidermidis strain FDAARGOS_913 plasmid unnamed6	100%	100%	64.4	7.00E-08	CP065660.1	MGE	Plasmid	This study
		Staphylococcus epidermidis plasmid SAP106A	100%	100%	64.4	7.00E-08	GQ900454.2	MGE	Plasmid	This study
87	TAAGGAACGAAATACTTTCATCATGATAAATAAAT	Staphylococcus xylosus strain 2 plasmid unnamed1	100%	91%	51.0	1.00E-03	CP031276.1	MGE	Plasmid	This study
88	ACTGTTTTTCGTTCGATTTCAAAGTCGATTTCATTA	Staphylococcus epidermidis strain SESURV_p2_0614 plasmid unnamed2	94%	97%	58.1	1.00E-05	CP043790.1	MGE	Plasmid	This study
		Mammaliicoccus sciuri strain C2865 plasmid pUR2865-2	94%	94%	53.6	2.00E-04	CP080325.1	MGE	Plasmid	This study
		Enterococcus faecalis strain NS2 plasmid pNS2A	94%	94%	53.6	2.00E-04	CP078163.1	MGE	Plasmid	This study
89	ACAAAGCAGTTTTTTGAACATGCTAAAACGTTTTTAG	Staphylococcus aureus P2D1C1 plasmid unnamed3	100%	100%	68.0	7.00E-09	CP033985.1	MGE	Plasmid	This study
		Staphylococcus aureus strain P2D8C1 plasmid unnamed3	100%	100%	68.0	7.00E-09	CP033980.1	MGE	Plasmid	This study
		Staphylococcus aureus strain P2D15C1 plasmid unnamed3	100%	100%	68.0	7.00E-09	CP033975.1	MGE	Plasmid	This study
90	ATTGAAATTTGAAACATTATTTGGGGGTGTAAGT	SL chromosome	NA	NA	NA	NA	NA	SL chromosome	NA	This study
91	ACATTTAGCAGATAATCGTGCAGCACATGATTTTG	Staphylococcus phage IME1323_01	100%	94%	55.0	3.00E-05	KY653117.1	MGE	Phage	This study
		Staphylococcus phage StB12	100%	94%	55.0	3.00E-05	JN700520.2	MGE	Phage	This study
92	TATAAATCTTTTTTAAAACCTTTTTAGGACTCAAATG	Staphylococcus hominis strain FDAARGOS_747 plasmid unnamed2	75%	100%	51.8	5.00E-04	CP046302.1	MGE	Plasmid	This study
		Staphylococcus epidermidis strain SESURV_p3_1362 plasmid unnamed1	89%	94%	48.2	7.00E-03	CP043802.1	MGE	Plasmid	This study
		Staphylococcus epidermidis strain SESURV_p3_0825 plasmid unnamed2	89%	94%	48.2	7.00E-03	CP043794.1	MGE	Plasmid	This study
93	GTGTTTCTTCAAAATGCCCCTTTAAAATTAAAAATA	Staphylococcus simulans strain MR1 plasmid unnamed1	100%	100%	66.2	2.00E-08	CP015643.1	MGE	Plasmid	This study
		Staphylococcus simulans strain MR2 plasmid unnamed1	100%	100%	66.2	2.00E-08	CP016158.1	MGE	Plasmid	This study
		Staphylococcus simulans strain MR4 plasmid unnamed4	100%	100%	66.2	2.00E-08	CP017431.1	MGE	Plasmid	This study
94	CATGTTTTTGCTCCATTTCTATAATTTTTTGTATATA	Staphylococcus lugdunensis SL13 prophage (Staphylococcus phage 187-like)	100%	100%	68.0	7.00E-09	CP041722.1	MGE	Phage	This study
		Staphylococcus lugdunensis SL55 prophage (Staphylococcus phage phiETA2-lke)	100%	100%	68.0	7.00E-09	CP041724.1	MGE	Phage	This study
		Staphylococcus lugdunensis SL117 prophage (Staphylococcus phage StB12-like)	100%	100%	68.0	7.00E-09	CP041725.1	MGE	Phage	This study
95	TTAATTTTTCTTTGATTTGTTCTTTTGTGCTTTTTT	None	NA	NA	NA	NA	NA	None	NA	This study
96	ACCATTGTTAATTATCTCCCTCGAAAAAGTAGTGACC	Staphylococcus lugdunensis SL122 prophage (Staphylococcus phage StB12-like)	100%	97%	63.5	3.00E-07	CP041727.1	MGE	Phage	This study
		Staphylococcus lugdunensis VISLISI_33 prophage (Staphylococcus phage PH15-like)	100%	97%	63.5	3.00E-07	CP020769.1	MGE	Phage	This study
		Staphylococcus lugdunensis VISLISI_37 prophage (Staphylococcus phage StB12-like)	100%	97%	63.5	3.00E-07	CP020761.1	MGE	Phage	This study
97	CGACATTAAATATTTCATAATCAAAATCTGTTTTT	Vibrio fischeri MJ11 plasmid pMJ100	85%	94%	44.6	4.70E-02	CP001134.1	MGE	Plasmid	This study
98	CATTCACTCTAAGTATATTTAACAAATCAATATATT	Staphylococcus haemolyticus strain 12b plasmid pSH_12b_2	100%	100%	66.2	2.00E-08	CP071507.1	MGE	Plasmid	This study
		Staphylococcus haemolyticus strain 7b plasmid pSH_7b_3, complete sequence	100%	100%	66.2	2.00E-08	CP071511.1	MGE	Plasmid	This study
		Staphylococcus haemolyticus strain 1b plasmid pSH_1b_3, complete sequence	100%	100%	66.2	2.00E-08	CP071515.1	MGE	Plasmid	This study
99	CTTCAACCATTTCATCATCTTTTCTCATTTCTTCA	Staphylococcus phage Pontiff	100%	94%	55.4	3.00E-05	MH972262.1	MGE	Phage	This study
		Staphylococcus phage SeAlphi	94%	91%	47.3	1.80E-02	MZ152915.1	MGE	Phage	This study
100	AGCACTTTTTTTCTTTTAGCTCGATATTCTGGA	Staphylococcus lugdunensis SL117 prophage (Staphylococcus phage StB12-like)	93%	100%	57.2	1.00E-05	CP041725.1	MGE	Phage	This study
		Staphylococcus lugdunensis SL122 prophage (Staphylococcus phage StB12-like)	93%	100%	57.2	1.00E-05	CP041727.1	MGE	Phage	This study
		Staphylococcus lugdunensis VISLISI_33 prophage (Staphylococcus phage PH15-like)	93%	100%	57.2	1.00E-05	CP020769.1	MGE	Phage	This study
101	GTTCTAAGTGTTGCATCTACGATTTGATTTATTT	None	NA	NA	NA	NA	NA	None	NA	This study
102	CTATACTTAAAAACACTGTCTAACAATTCTAGTTT	Staphylococcus lugdunensis SL13 prophage (Staphylococcus phage 187-like)	100%	100%	64.4	7.00E-08	CP041722.1	MGE	Phage	This study
		Staphylococcus lugdunensis SL117 prophage (Staphylococcus phage StB12-like)	100%	100%	64.4	7.00E-08	CP041725.1	MGE	Phage	This study
		Staphylococcus lugdunensis SL122 prophage (Staphylococcus phage StB12-like)	100%	100%	64.4	7.00E-08	CP041727.1	MGE	Phage	This study
103	GCGATTGGTAACTCATATCCGTTTGTAATCTGTGTACT	Staphylococcus lugdunensis SL13 prophage (Staphylococcus phage 187-like)	100%	100%	73.4	3.00E-10	CP041722.1	MGE	Phage	This study
		Staphylococcus lugdunensis FDAARGOS_143 prophage (Staphylococcus phage CNPx-like)	100%	100%	73.4	3.00E-10	CP014023.2	MGE	Phage	This study
		Bacteriophage 85	95%	89%	51.8	1.00E-03	AY954953.1	MGE	Phage	This study
104	GGGCGTTTTGTTCGTCGCGTTGTTTGAGTTTTTGTT	Staphylococcus lugdunensis VISLISI_33 prophage (Staphylococcus phage PH15-like)	100%	94%	57.2	1.00E-05	CP020769.1	MGE	Phage	This study
		Staphylococcus phage HOB 14.1.R1	80%	100%	53.6	2.00E-04	CP018841.1	MGE	Phage	This study
105	ATTTGTCCCTCTCTCACATGTCTCTCAATAAATTTT	Staphylococcus simulans bv. staphylolyticus strain NRRL B-2628 plasmid pACK2	100%	100%	66.2	2.00E-08	HQ170520.3	MGE	Plasmid	This study
		Staphylococcus simulans strain MR1 plasmid unnamed1	100%	97%	61.7	1.00E-06	CP015643.1	MGE	Plasmid	This study
		Staphylococcus simulans strain MR2 plasmid unnamed1	100%	97%	61.7	1.00E-06	CP016158.1	MGE	Plasmid	This study
106	ACCTCCTTAAAAGGATTTAAATAAATACTGTTTTTTC	None	NA	NA	NA	NA	NA	None	NA	This study
107	TTATATTCTTTTGTAGATATATCGATTTTTTGTCTTAT	None	NA	NA	NA	NA	NA	None	NA	This study
108	ATGTCTAAATAAGTATGTATTACATAGAATTATGTG	Staphylococcus aureus strain 14640 plasmid unnamed	100%	100%	66.2	2.00E-08	CP053637.1	MGE	Plasmid	This study
		Staphylococcus aureus strain 14638 plasmid unnamed	100%	100%	66.2	2.00E-08	CP053635.1	MGE	Plasmid	This study
		Staphylococcus aureus strain UP_274 plasmid unnamed	100%	100%	66.2	2.00E-08	CP047853.1	MGE	Plasmid	This study
109	CGATTTATTAACAGCAACCAATACATGAAAATGCG	Staphylococcus aureus strain MJ015 plasmid unnamed1	91%	94%	50.0	1.00E-03	CP038184.1	MGE	Plasmid	This study
		Staphylococcus warneri strain WB224 plasmid pWB224_5	91%	94%	50.0	1.00E-03	CP053475.1	MGE	Plasmid	This study
		Staphylococcus aureus strain SR153 plasmid pSR02	91%	94%	50.0	1.00E-03	CP048645.1	MGE	Plasmid	This study
110	CAAAAGTTTTATTATATCTATCTTCTGCACTTTCTA	None	NA	NA	NA	NA	NA	None	NA	This study
111	TCAAAAATGACCATTGTTATAATTGAAATAAGTATG	Staphylococcus lugdunensis SL13 prophage (Staphylococcus phage 187-like)	100%	100%	66.2	2.00E-08	CP041722.1	MGE	Phage	This study
		Staphylococcus lugdunensis SL117 prophage (Staphylococcus phage StB12-like)	100%	100%	66.2	2.00E-08	CP041725.1	MGE	Phage	This study
		Staphylococcus lugdunensis SL122 prophage (Staphylococcus phage StB12-like)	100%	100%	66.2	2.00E-08	CP041727.1	MGE	Phage	This study
112	TGTATTGATTGCTAGTTTCTTGTGGCATTTCTTCGCT	Staphylococcus lugdunensis SL13 prophage (Staphylococcus phage 187-like)	100%	100%	68.0	7.00E-09	CP041722.1	MGE	Phage	This study
		Staphylococcus lugdunensis FDAARGOS_143 prophage (Staphylococcus phage CNPx-like)	100%	100%	68.0	7.00E-09	CP014023.2	MGE	Phage	This study
113	CAATGAATCGCTCAATCATATTTTTATTTCTCCTTC	Staphylococcus lugdunensis SL117 prophage (Staphylococcus phage StB12-like)	100%	100%	66.2	2.00E-08	CP041725.1	MGE	Phage	This study
		Staphylococcus lugdunensis SL122 prophage (Staphylococcus phage StB12-like)	100%	100%	66.2	2.00E-08	CP041727.1	MGE	Phage	This study
		Staphylococcus phage HSA84	100%	100%	66.2	2.00E-08	MG557619.1	MGE	Phage	This study
114	ACTTTTTCAACTTCTTGCACAATTCTGTCCCATTCATA	Staphylococcus lugdunensis SL55 prophage (Staphylococcus phage phiETA2-lke)	100%	100%	69.8	3.00E-09	CP041724.1	MGE	Phage	This study
		Staphylococcus lugdunensis SL13 prophage (Staphylococcus phage 187-like)	100%	100%	69.8	3.00E-09	CP041722.1	MGE	Phage	This study
		Staphylococcus lugdunensis SL118 prophage (Staphylococcus phage StB12-like)	100%	100%	69.8	3.00E-09	CP041726.1	MGE	Phage	This study
115	TTAAATTCTCAAGTTGTTTATTGATACGATCAGGAA	None	NA	NA	NA	NA	NA	None	NA	This study
116	TCTATAATTTTATTTAATGCTTTAGAAGCTTTAA	Staphylococcus lugdunensis SL13 prophage (Staphylococcus phage 187-like)	100%	100%	62.6	2.00E-07	CP041722.1	MGE	Phage	This study
		Staphylococcus lugdunensis FDAARGOS_143 prophage (Staphylococcus phage CNPx-like)	100%	97%	58.1	1.00E-05	CP014023.2	MGE	Phage	This study
117	TGTTGTTTATTAAAATAATTAGTATTCATAATTTGTTT	None	NA	NA	NA	NA	NA	None	NA	This study
118	CAATAAAACGTGCATATGTTGATTATAACTTCC	Staphylococcus epidermidis strain none genome assembly, plasmid: 3	100%	100%	60.8	8.00E-07	LR735439.1	MGE	Plasmid	This study
		Staphylococcus aureus strain ER03544.3 plasmid unnamed1	100%	100%	60.8	8.00E-07	CP030631.1	MGE	Plasmid	This study
		Staphylococcus aureus strain ER03556.3 plasmid unnamed1	100%	100%	60.8	8.00E-07	CP030648.1	MGE	Plasmid	This study
119	TATCAAAGCTAGCGTTTTTTTATGCTTTTTTGCCCCTC	Staphylococcus epidermidis strain none genome assembly, plasmid: 3	100%	100%	77.0	3.00E-11	LR735439.1	MGE	Plasmid	This study
		Staphylococcus aureus strain ER03544.3 plasmid unnamed1	100%	100%	77.0	3.00E-11	CP030631.1	MGE	Plasmid	This study
		Staphylococcus aureus strain ER03556.3 plasmid unnamed1	100%	100%	77.0	3.00E-11	CP030648.1	MGE	Plasmid	This study
120	CTTTGTCATGCAATAAATATATTTTTTCATTCATTTA	Staphylococcus epidermidis strain HD75-1 plasmid pHD75-1_1	97%	94%	57.2	1.00E-05	CP052942.1	MGE	Plasmid	This study
121	TGTTAAGTTTTTCTTACCATAATCATGAATATTGCTCA	None	NA	NA	NA	NA	NA	None	NA	This study
122	TAGAGAAAGGTGAGTGTTAATGATGACGATATTAGCA	Staphylococcus caprae strain SY333 plasmid pSY333-41	100%	100%	68.0	7.00E-09	CP051645.1	MGE	Plasmid	This study
		Staphylococcus aureus strain B3-14B plasmid pSALNT46	100%	100%	68.0	7.00E-09	CP042004.1	MGE	Plasmid	This study
		Staphylococcus saprophyticus subsp. saprophyticus ATCC 15305 = NCTC 7292 strain ATCC 15305 plasmid unnamed2	100%	100%	68.0	7.00E-09	CP035296.1	MGE	Plasmid	This study

Table S5. Sequence matches of spacer from type IIC CRISPR-Cas systems to NCBI non-redundant database

Spacer	Sequence	BLAST match with known MGEs	Query Cover	Identity %	Max score	E-value	Accession no.	Location	MGE	Reference
1	TGTTCGTCCATTTGTCTTTAAAATTATAT	None	NA	NA	NA	NA	NA	None	NA	This study
2	TTGGTGGCGGAGCTATCATTGCAGTAGCAG	Staphylococcus warneri strain 16A plasmid unnamed1	100%	97%	52.0	1.00E-03	CP031270.1	MGE	plasmid	This study
		Staphylococcus caprae strain SY333 plasmid pSY333-92	73%	100%	44.1	2.50E-01	CP051644.1	MGE	plasmid	This study
		Bacillus phage B5S	66%	100%	40.1	4.00E+00	NC 047736.1	MGE	phage	This study
3	GTATAAAAAAACACCGTATCAACTAAGAT	None	NA	NA	NA	NA	NA	None	NA	This study
4	ATTATGACATCAATAGTATCACTTTCCCAG	Staphylococcus lugdunensis SL55 prophage (Staphylococcus phage phiETA2-lke)	100%	100%	60.0	4.00E-06	CP041724.1	MGE	phage	This study
		Staphylococcus lugdunensis SL118 prophage (Staphylococcus phage StB12-like)	100%	100%	60.0	4.00E-06	CP041726.1	MGE	phage	This study
5	TGAAGGTAAAGACAGAGGACATAGTCCTA	None	NA	NA	NA	NA	NA	None	NA	This study
6	CGGTTTAGGTGCTGTTGCAATGGCTGGAA	Staphylococcus lugdunensis FDAARGOS 143 prophage (Staphylococcus phage CNPx-like)	96%	100%	56.0	4.00E-05	CP014023.2	MGE	phage	This study
		Staphylococcus lugdunensis FDAARGOS 117 prophage (Staphylococcus phage CNPx-like)	96%	96%	48.1	1.40E-02	CP041725.1	MGE	phage	This study
7	GTTTTTCGAATGAGTTGATAGTGACGTCTG	None	NA	NA	NA	NA	NA	None	NA	This study
8	TCACATCATTCGAACGTAAGAATGAGGCTG	Staphylococcus lugdunensis SL55 prophage (Staphylococcus phage phiETA2-lke)	100%	100%	60.0	4.00E-06	CP041724.1	MGE	phage	This study
		Staphylococcus lugdunensis SL118 prophage (Staphylococcus phage StB12-like)	100%	100%	60.0	4.00E-06	CP041726.1	MGE	phage	This study
		Staphylococcus lugdunensis SL13 prophage (Staphylococcus phage 187-like)	100%	97%	52.0	1.00E-03	CP041722.1	MGE	phage	This study
9	TCTGTGTTCAATCCATGCAGTCTGTCCAA	None	NA	NA	NA	NA	NA	None	NA	This study
10	GTATTGAAGATGTAAGAAAAGACACGGTTC	None	NA	NA	NA	NA	NA	None	NA	This study
11	ACTAAAAACTGACTGCTACGGCAGAGATCA	Staphylococcus lugdunensis VISLSI 22 prophage (Staphylococcus phage StB12-like)	93%	100%	56.0	7.00E-05	CP020764.1	MGE	phage	This study
		Staphylococcus lugdunensis N920143 prophage (Staphylococcus phage TEM123-like)	93%	100%	56.0	7.00E-05	FR870271.1	MGE	phage	This study
		Staphylococcus lugdunensis SL122 prophage (Staphylococcus phage StB12-like)	86%	100%	52.0	1.00E-03	CP041727.1	MGE	phage	This study
12	TAAGAAAACGCCAATTATTGTTGTAGTAA	None	NA	NA	NA	NA	NA	None	NA	This study
13	CGCCTTTTCCAAGTGCGGTAGTTGTATATC	None	NA	NA	NA	NA	NA	None	NA	This study
14	ACCCACCCATTTTTGACCGTCGTAATAAAA	Staphylococcus lugdunensis FDAARGOS 143 prophage (Staphylococcus phage CNPx-like)	100%	97%	52.0	1.00E-03	CP014023.2	MGE	phage	This study
		Staphylococcus lugdunensis SL117 prophage (Staphylococcus phage CNPx-like)	100%	97%	52.0	1.00E-03	CP041725.1	MGE	phage	This study
15	GCCCTATATATCTCGTTCTAATCGCAGTGT	Staphylococcus lugdunensis SL117 prophage (Staphylococcus phage CNPx-like)	93%	100%	56.0	7.00E-05	CP041725.1	MGE	phage	This study
		Staphylococcus lugdunensis HKU09-01 prophage (Staphylococcus phage PH15-like)	93%	100%	56.0	7.00E-05	CP001837.1	MGE	phage	This study
		Staphylococcus lugdunensis FDAARGOS 143 prophage (Staphylococcus phage CNPx-like)	83%	96%	42.1	1.00E+00	CP014023.2	MGE	phage	This study
16	TGAATTATTTGACCCAATCGCATTAGGTAA	Staphylococcus phage St 134	100%	100%	60.0	4.00E-06	NC 047814.1	MGE	phage	This study
		Staphylococcus phage JBug18	100%	93%	44.1	2.50E-01	MH972263.1	MGE	phage	This study
		Staphylococcus phage Pontiff	100%	93%	44.1	2.50E-01	MH972262.1	MGE	phage	This study
17	GAAATACACAAGATTATATTGCAGTGGGTT	Staphylococcus phage JBug18	100%	97%	52.0	1.00E-03	MH972263.1	MGE	phage	This study
		Staphylococcus phage Pontiff	100%	97%	52.0	1.00E-03	MH972262.1	MGE	phage	This study
		Staphylococcus phage Pike	100%	97%	52.0	1.00E-03	MH972261.1	MGE	phage	This study
18	GAATTGACTGAAGATATTGACCGACGAATA	Staphylococcus phage JBug18	100%	100%	60.0	4.00E-06	MH972263.1	MGE	phage	This study
		Staphylococcus phage Pontiff	100%	100%	60.0	4.00E-06	MH972262.1	MGE	phage	This study
		Staphylococcus phage Pike	100%	100%	60.0	4.00E-06	MH972261.1	MGE	phage	This study
19	GGGTTCGAGTGCATAGAACGATTTCTATAA	None	NA	NA	NA	NA	NA	None	NA	This study
20	ATTTGGATTTGTTTCAAAGAATTTAGGCAT	Staphylococcus pseudintermedius strain ME4692 plasmid unnamed	96%	93%	42.1	1.00E+00	CP039748.1	MGE	plasmid	This study
21	TAAGTTCGTTGGCATTGTTCGTGGAGTTG	Staphylococcus lugdunensis SL55 prophage (Staphylococcus phage phiETA2-lke)	86%	96%	42.1	8.90E-01	CP041724.1	MGE	phage	This study
		Staphylococcus lugdunensis SL118 prophage (Staphylococcus phage StB12-like)	86%	96%	42.1	8.90E-01	CP041726.1	MGE	phage	This study
		Staphylococcus lugdunensis HKU09-01 prophage (Staphylococcus phage PH15-like)	86%	96%	42.1	8.90E-01	CP001837.1	MGE	phage	This study
22	TGCAAAAAGACGTTTACCTATATCATATTT	None	NA	NA	NA	NA	NA	None	NA	This study
23	TTGTTTTGCGTCTGCGTCATCTTCATCAG	Staphylococcus phage phiSa2wa_st59	100%	100%	58.0	1.00E-05	MG029512.1	MGE	phage	This study
		Staphylococcus phage BUCT556	100%	100%	58.0	1.00E-05	MW554488.1	MGE	phage	This study
		Staphylococcus phage phiSa2wa_st	100%	100%	58.0	1.00E-05	NC_055046.1	MGE	phage	This study
24	CGTCGTAATAAAAAGGTGCATATATACCTT	Staphylococcus lugdunensis FDAARGOS_143 prophage (Staphylococcus phage CNPx-like)	100%	97%	52.0	1.00E-03	CP014023.2	MGE	phage	This study
		Staphylococcus lugdunensis SL117 prophage (Staphylococcus phage CNPx-like)	100%	97%	52.0	1.00E-03	CP041725.1	MGE	phage	This study
25	GCCAAATCATCTCTCTATCTACGAAAACGT	Staphylococcus lugdunensis SL117 prophage (Staphylococcus phage CNPx-like)	100%	100%	60.0	4.00E-06	CP041725.1	MGE	phage	This study
		Staphylococcus lugdunensis FDAARGOS_143 prophage (Staphylococcus phage CNPx-like)	100%	100%	60.0	4.00E-06	CP014023.2	MGE	phage	This study
		Halorussus sp. RC-68 plasmid unnamed2	66%	100%	40.1	4.00E+00	CP035121.1	MGE	plasmid	This study
26	AAATCCGGGGAATATATCACTATAATGGAT	Staphylococcus phage JBug18	66%	100%	40.1	4.00E+00	MH972263.1	MGE	phage	This study
		Staphylococcus phage SeAlphi	66%	100%	40.1	4.00E+00	MZ152915.1	MGE	phage	This study
		Staphylococcus phage Pontiff	66%	100%	40.1	4.00E+00	MH972262.1	MGE	phage	This study
27	GTATCATCTATATAGTTAACGTCTTTTATA	None	NA	NA	NA	NA	NA	None	NA	This study
28	ACAGGCGATTTAACATCTAACATTATTAGA	None	NA	NA	NA	NA	NA	None	NA	This study
29	GATAAAGAATGTTTTTTACACATATGGTAG	Staphylococcus phage JBug18	100%	100%	60.0	4.00E-06	MH972263.1	MGE	phage	This study
		Staphylococcus phage Pontiff	100%	100%	60.0	4.00E-06	MH972262.1	MGE	phage	This study
		Staphylococcus phage Pike	100%	100%	60.0	4.00E-06	MH972261.1	MGE	phage	This study
30	TCGGCATATTTATTGTTGACATATTGCCAA	None	NA	NA	NA	NA	NA	None	NA	This study
31	TTAGTCGTATTGATAGCCAAGCTGTATTCC	Staphylococcus lugdunensis SL117 prophage (Staphylococcus phage CNPx-like)	100%	97%	52.0	1.00E-03	CP041725.1	MGE	phage	This study
		Staphylococcus lugdunensis FDAARGOS_143 prophage (Staphylococcus phage CNPx-like)	100%	97%	52.0	1.00E-03	CP014023.2	MGE	phage	This study
		Staphylococcus phage PMBT9	93%	93%	40.1	4.00E+00	MW221967.1	MGE	phage	This study
32	GAATACCACCACCTAAAATTCTATCATATT	Staphylococcus phage KSAP11	100%	97%	52.0	1.00E-03	LC492752.1	MGE	phage	This study
		Staphylococcus phage KSAP7	100%	97%	52.0	1.00E-03	LC492751.1	MGE	phage	This study
		Staphylococcal phage MR003	100%	97%	52.0	1.00E-03	AP019522.1	MGE	phage	This study
33	CTAGCAAAAAGCGTAATCCTCAAAAAGGAA	None	NA	NA	NA	NA	NA	None	NA	This study
34	GTGGGGTTGTTGAAAAAACTTTCGGATCAG	Dolichospermum sp. UHCC 0315A plasmid pUHCC0315c	80%	96%	40.1	4.00E+00	CP043059.1	MGE	plasmid	This study
		Dolichospermum sp. UHCC 0315A plasmid pUHCC0315b	80%	96%	40.1	4.00E+00	CP043058.1	MGE	plasmid	This study
		Dolichospermum sp. UHCC 0315A plasmid pUHCC0315a	80%	96%	40.1	4.00E+00	CP043057.1	MGE	plasmid	This study
35	AAAATTGAGAAGAATAAAAAATATGTTTT	Staphylococcus lugdunensis SL55 prophage (Staphylococcus phage phiETA2-lke)	100%	100%	58.0	1.00E-05	CP041724.1	MGE	phage	This study
		Staphylococcus lugdunensis SL118 prophage (Staphylococcus phage StB12-like)	100%	100%	58.0	1.00E-05	CP041726.1	MGE	phage	This study
36	AATCAATTTAATGATACTGAAATTGAATAT	None	NA	NA	NA	NA	NA	None	NA	This study
37	ACACGTTGACCGTTTGTGACTTGTCCGTCG	Staphylococcus phage JBug18	76%	100%	46.1	6.40E-02	MH972263.1	MGE	phage	This study
		Staphylococcus phage Pontiff	76%	100%	46.1	6.40E-02	MH972262.1	MGE	phage	This study
		Staphylococcus phage Pike	76%	100%	46.1	6.40E-02	MH972261.1	MGE	phage	This study
38	GTTCAGGTTTTGGTTTAGGTTGATTGTTC	None	NA	NA	NA	NA	NA	None	NA	This study
39	TTCTTTAGAAATACACAAGATTATATAGC	Staphylococcus phage JBug18	89%	100%	52.0	1.00E-03	MH972263.1	MGE	phage	This study
		Staphylococcus phage Pontiff	89%	100%	52.0	1.00E-03	MH972262.1	MGE	phage	This study
		Staphylococcus phage Pike	89%	100%	52.0	1.00E-03	MH972261.1	MGE	phage	This study
40	CAGACGATAGTGAAGGTGGTGCAGATAAAG	Catenovulum sp. CCB-QB4 plasmid unnamed1	66%	100%	40.1	4.00E+00	CP026605.1	MGE	plasmid	This study
41	TCTTTCACATCACTCATTGTTGCACTACTG	None	NA	NA	NA	NA	NA	None	NA	This study
42	ATATTTAGCAGGTGATAATGGATTAGACCAA	None	NA	NA	NA	NA	NA	None	NA	This study
43	TTAATAAAATATTAATTACAGAAGCACCA	Staphylococcus phage JBug18	100%	100%	58.0	1.00E-05	MH972263.1	MGE	phage	This study
		Staphylococcus phage SeAlphi	100%	100%	58.0	1.00E-05	MZ152915.1	MGE	phage	This study
		Staphylococcus phage St 134	100%	100%	58.0	1.00E-05	NC_047814.1	MGE	phage	This study
44	AAATAAGTTTTAAATAATGATTTTTCTGTA	None	NA	NA	NA	NA	NA	None	NA	This study
45	ACAGTGCATTTTATGCTTTAAGCCGCTTT	Staphylococcus lugdunensis SL117 prophage (Staphylococcus phage StB12-like)	100%	93%	42.1	8.90E-01	CP041725.1	MGE	phage	This study
		Staphylococcus lugdunensis SL122 prophage (Staphylococcus phage StB12-like)	100%	93%	42.1	8.90E-01	CP041727.1	MGE	phage	This study
		Staphylococcus lugdunensis VISLISI_22 prophage (Staphylococcus phage StB12-like)	100%	93%	42.1	8.90E-01	CP020764.1	MGE	phage	This study
46	CTATCAAGAGGGTGTAGTTGCAGGATTAA	None	NA	NA	NA	NA	NA	None	NA	This study
47	ATCTTGAACAAATGTCATGGCAAGACGCAC	Staphylococcus phage JBug18	100%	100%	60.0	4.00E-06	MH972263.1	MGE	phage	This study
		Staphylococcus phage Pontiff	100%	100%	60.0	4.00E-06	MH972262.1	MGE	phage	This study
		Staphylococcus phage Pike	100%	100%	60.0	4.00E-06	MH972261.1	MGE	phage	This study
48	AATATAATAATCAACAAAATATGTATGGTG	None	NA	NA	NA	NA	NA	None	NA	This study
49	CAGATACATTAAATGTAAATACTGAACCAG	Staphylococcus phage JBug18	100%	100%	60.0	4.00E-06	MH972263.1	MGE	phage	This study
		Staphylococcus phage Pike	100%	100%	60.0	4.00E-06	MH972261.1	MGE	phage	This study
		Staphylococcus phage Andhra	100%	100%	60.0	4.00E-06	NC_047813.1	MGE	phage	This study
50	TTGTGGTGAAATTGGTATACACTTCTCAC	Staphylococcus phage JBug18	100%	97%	50.1	4.00E-03	MH972263.1	MGE	phage	This study
		Staphylococcus phage SeAlphi	100%	97%	50.1	4.00E-03	MZ152915.1	MGE	phage	This study
		Staphylococcus phage Pontiff	100%	97%	50.1	4.00E-03	MH972262.1	MGE	phage	This study
51	AAGCCTTGTTGGTTCCAAACCATAAGGCAA	Staphylococcus aureus strain UP_1278 plasmid unnamed1	100%	100%	60.0	4.00E-06	CP047823.1	MGE	plasmid	This study
		Staphylococcus aureus strain RIVM6519 plasmid pRIVM6519-2	100%	100%	60.0	4.00E-06	CP015175.1	MGE	plasmid	This study
		Staphylococcus aureus strain NL1 plasmid pNL1-02	100%	100%	60.0	4.00E-06	CP077743.1	MGE	plasmid	This study
52	ATTCTAAATCATCAATATTAATAAAGTTGG	Staphylococcus phage JBug18	83%	100%	50.1	4.00E-03	MH972263.1	MGE	phage	This study
		Staphylococcus phage Pontiff	83%	100%	50.1	4.00E-03	MH972262.1	MGE	phage	This study
		Staphylococcus phage Pike	83%	100%	50.1	4.00E-03	MH972261.1	MGE	phage	This study
53	TTATAGAATACCTGAACGACCAATTCAAAC	Staphylococcus lugdunensis VISLSI_33 prophage (Staphylococcus phage PH15-like)	100%	97%	52.0	1.00E-03	CP020769.1	MGE	phage	This study
		Staphylococcus lugdunensis SL55 prophage (Staphylococcus phage phiETA2-lke)	93%	96%	48.1	1.60E-02	CP041724.1	MGE	phage	This study
		Staphylococcus lugdunensis SL118 prophage (Staphylococcus phage StB12-like)	93%	96%	48.1	1.60E-02	CP041726.1	MGE	phage	This study
54	CGGTTATAGTTATTACTGCCTATATTTTAC	None	NA	NA	NA	NA	NA	None	NA	This study
55	CCCTCTAAGACAATATCACCCTCACGTTTA	Secundilactobacillus paracollinoides strain TMW 1.1979 plasmid pL11979-2	66%	100%	40.1	4.00E+00	CP014914.1	MGE	plasmid	This study
		Pediococcus damnosus strain TMW 2.1533 plasmid pL21533-4	66%	100%	40.1	4.00E+00	CP012279.1	MGE	plasmid	This study
56	TTGGTTTTGTTGCGTCTTGTTCACCTTTAT	None	NA	NA	NA	NA	NA	None	NA	This study
57	AAATACGCAAGATTATATTGCAGTGGGTT	Staphylococcus phage St 134	100%	100%	58.0	1.00E-05	NC_047814.1	MGE	phage	This study
		Staphylococcus phage SeAlphi	100%	100%	58.0	1.00E-05	MZ152915.1	MGE	phage	This study
		Staphylococcus phage JBug18	100%	93%	42.1	8.90E-01	MH972263.1	MGE	phage	This study
58	TATTAGTAATAAATTTCGATATGAATAGCT	Staphylococcus epidermidis strain SESURV_p1_1200 plasmid unnamed3	100%	100%	60.0	4.00E-06	CP043799.1	MGE	plasmid	This study
		Staphylococcus epidermidis strain SESURV_p1_0557 plasmid unnamed4	100%	100%	60.0	4.00E-06	CP043779.1	MGE	plasmid	This study
		Staphylococcus epidermidis strain CDC120 plasmid pSTA491	100%	100%	60.0	4.00E-06	CP034112.1	MGE	plasmid	This study
59	TTTTGGCATAACGACGGAACGAACAATCCA	Staphylococcus phage St 134	100%	100%	60.0	4.00E-06	NC_047814.1	MGE	phage	This study
		Staphylococcus phage SeAlphi	100%	97%	52.0	1.00E-03	MZ152915.1	MGE	phage	This study
		Staphylococcus phage JBug18	100%	97%	52.0	1.00E-03	MH972263.1	MGE	phage	This study
60	AAAAGTGAATTGTGTTTTGATAATCTGTCA	Staphylococcus phage JBug18	100%	100%	60.0	4.00E-06	MH972263.1	MGE	phage	This study
		Staphylococcus phage Pontiff	100%	100%	60.0	4.00E-06	MH972262.1	MGE	phage	This study
		Staphylococcus phage Pike	100%	100%	60.0	4.00E-06	MH972261.1	MGE	phage	This study
61	ATAGCGATGATTCAGTACAAGAAGTATAG	Staphylococcus phage JBug18	100%	97%	50.1	4.00E-03	MH972263.1	MGE	phage	This study
		Staphylococcus phage Pontiff	100%	97%	50.1	4.00E-03	MH972262.1	MGE	phage	This study
		Staphylococcus phage Pike	100%	97%	50.1	4.00E-03	MH972261.1	MGE	phage	This study
62	CGAAGTCGAATAGCATTTGTTGAATGTGTG	Staphylococcus lugdunensis VISLSI_33 prophage (Staphylococcus phage PH15-like)	100%	100%	60.0	4.00E-06	CP020769.1	MGE	phage	This study
		Staphylococcus phage WBG8381	93%	100%	56.0	7.00E-05	CP071049.1	MGE	phage	This study
		Staphylococcus phage IME1364_02	93%	100%	56.0	7.00E-05	MW238767.1	MGE	phage	This study
63	ATTTCAGATAAATTTAATGTGTCTAATCAA	None	NA	NA	NA	NA	NA	None	NA	This study
64	CTTCGCCCTCAGGTTGTTGTGTGTCATCTT	None	NA	NA	NA	NA	NA	None	NA	This study
65	TCGACCTTTTGTCAAATAGACATCGTGTAC	None	NA	NA	NA	NA	NA	None	NA	This study
66	AACAAGGCCTTAGAGGCGCAGAATTGATTT	Staphylococcus lugdunensis SL55 prophage (Staphylococcus phage phiETA2-lke)	100%	100%	60.0	4.00E-06	CP041724.1	MGE	phage	This study
		Staphylococcus lugdunensis SL118 prophage (Staphylococcus phage StB12-like)	100%	100%	60.0	4.00E-06	CP041726.1	MGE	phage	This study
		Staphylococcus lugdunensis SL122 prophage (Staphylococcus phage StB12-like)	100%	93%	44.1	2.60E-01	CP041727.1	MGE	phage	This study
67	GATAATCTAGCTTGTACTGTTGAGCCGTGT	None	NA	NA	NA	NA	NA	None	NA	This study
68	TTAGTACACATATTAGGTCATTAATTGCAC	None	NA	NA	NA	NA	NA	None	NA	This study
69	AAGTGAGTTTAATAAGCGCAAGTGACGAAA	None	NA	NA	NA	NA	NA	None	NA	This study
70	ATGATGGATTACGTAAACATATTTCAAGCA	None	NA	NA	NA	NA	NA	None	NA	This study
71	ATATGGACGAAAAAACACCTCATATGCATT	Staphylococcus aureus strain P2D1C1 plasmid unnamed3	100%	100%	60.0	4.00E-06	CP033985.1	MGE	plasmid	This study
		Staphylococcus aureus strain P2D8C1 plasmid unnamed3	100%	100%	60.0	4.00E-06	CP033980.1	MGE	plasmid	This study
		Staphylococcus aureus strain P2D15C1 plasmid unnamed3	100%	100%	60.0	4.00E-06	CP033975.1	MGE	plasmid	This study
72	TCGTTTCAGATTTATTATTTTGTTGTGTAG	Bacteriophage sp. isolate 165	66%	100%	40.1	3.10E+00	MN855824.1	MGE	phage	This study
73	ATTTTCTATTATTGCAATGTGGAATTGGGA	Enterococcus faecalis strain L8 plasmid pL8	100%	100%	60.0	4.00E-06	CP042217.2	MGE	plasmid	This study
		Staphylococcus aureus strain HL17078 plasmid pHL17078_1	100%	100%	60.0	4.00E-06	CP080557.1	MGE	plasmid	This study
		Macrococcus caseolyticus strain 19Msa0287 plasmid p19Msa0287-3	100%	100%	60.0	4.00E-06	CP079953.1	MGE	plasmid	This study
74	AAACTGTAATAGAAGATATCGCAGAAGAGT	Staphylococcus lugdunensis SL55 prophage (Staphylococcus phage phiETA2-lke)	96%	100%	58.0	2.00E-05	CP041724.1	MGE	phage	This study
		Staphylococcus lugdunensis SL118 prophage (Staphylococcus phage StB12-like)	96%	100%	58.0	2.00E-05	CP041726.1	MGE	phage	This study
		Staphylococcus lugdunensis SL13 prophage (Staphylococcus phage 187-like)	93%	96%	48.1	1.60E-02	CP041722.1	MGE	phage	This study
75	TCATCTATTTCAGATTGTGTTAGAGACTTT	Staphylococcus phage Andhra	100%	100%	60.0	4.00E-06	NC_047813.1	MGE	phage	This study
		Staphylococcus phage SeAlphi	100%	100%	60.0	4.00E-06	MZ152915.1	MGE	phage	This study
		Staphylococcus phage St 134	100%	97%	52.0	1.00E-03	NC_047814.1	MGE	phage	This study
76	TTACTATAAGCATTATCAAAATGAAGATTT	Staphylococcus phage JBug18	100%	97%	52.0	1.00E-03	MH972263.1	MGE	phage	This study
		Staphylococcus phage Pontiff	100%	97%	52.0	1.00E-03	MH972262.1	MGE	phage	This study
		Staphylococcus phage Pike	100%	97%	52.0	1.00E-03	MH972261.1	MGE	phage	This study
77	TTATATAAGCCAAAAAGAATGGCTTGAACT	Staphylococcus aureus strain RIVM6519 plasmid pRIVM6519-2	100%	100%	60.0	4.00E-06	CP015175.1	MGE	plasmid	This study
		Staphylococcus warneri plasmid pSW49	100%	100%	60.0	4.00E-06	AM040730.1	MGE	plasmid	This study
		Staphylococcus warneri plasmid pSW174	100%	100%	60.0	4.00E-06	AM040729.1	MGE	plasmid	This study
78	AAGAAGATAAAAAAGTACGTGGGAATAAGT	Staphylococcus aureus strain tu2 plasmid pRIVM4390	100%	100%	60.0	4.00E-06	MH785256.1	MGE	plasmid	This study
		Staphylococcus aureus strain ch24 plasmid pRIVM4390	100%	100%	60.0	4.00E-06	MH785228.1	MGE	plasmid	This study
		Staphylococcus epidermidis strain 25S plasmid pLF25	100%	100%	60.0	4.00E-06	MH090916.1	MGE	plasmid	This study
79	TTTAACCATGATTATGGTAAGCAATGGACTT	Staphylococcus phage Andhra	100%	97%	52.7	4.00E-04	NC_047813.1	MGE	phage	This study
		Staphylococcus phage JBug18	100%	94%	48.2	5.00E-03	MH972263.1	MGE	phage	This study
		Staphylococcus phage Pontiff	100%	94%	48.2	5.00E-03	MH972262.1	MGE	phage	This study
80	GCACTGCATTTAGTGCTTTAACTGGTCCAG	Staphylococcus phage IME1365_01	100%	100%	60.0	4.00E-06	KY653129.1	MGE	phage	This study
81	TTATTCATCATAATTATTTTATTAAAACAC	Staphylococcus phage St 134	100%	100%	60.0	4.00E-06	NC_047814.1	MGE	phage	This study
82	TATTTCTAGCTACAACCACCATAATCAGTG	Staphylococcus aureus strain MJ015 plasmid unnamed1	100%	100%	60.0	4.00E-06	CP038184.1	MGE	plasmid	This study
		Staphylococcus warneri strain WB224 plasmid pWB224_2	100%	100%	60.0	4.00E-06	CP053472.1	MGE	plasmid	This study
		Staphylococcus aureus strain SR153 plasmid pSR02	100%	100%	60.0	4.00E-06	CP048645.1	MGE	plasmid	This study
83	CGCCGTAAGCATTGGGTCAAACCTTTCACCA	None	NA	NA	NA	NA	NA	None	NA	This study
84	CACTATTCCAAAAATAAACCACTGCACCAC	None	NA	NA	NA	NA	NA	None	NA	This study

References

1. Argemi X, Matelska D, Ginalski K, Riegel P, Hansmann Y, Bloom J, Pestel-Caron M, Dahyot S, Lebeurre J, Prevost G. 2018. Comparative genomic analysis of Staphylococcus lugdunensis shows a closed pan-genome and multiple barriers to horizontal gene transfer. BMC Genomics 19:621.
